# Supplementary material for: Arabidopsis conditional photosynthesis mutants abc1k1 and var2 accumulate partially processed thylakoid preproteins and are defective in chloroplast biogenesis
Source: Commun Biol. 2025 Jan 22;8:111. doi: 10.1038/s42003-025-07497-y (PMC11754785; doi:10.1038/s42003-025-07497-y)

Supplementary Figure 1

Uncropped and replicated blots on p. 1 to 26 correspond to Figure 1c, those on p. 27-30 to Figure 8c

PsbA Replicate 1

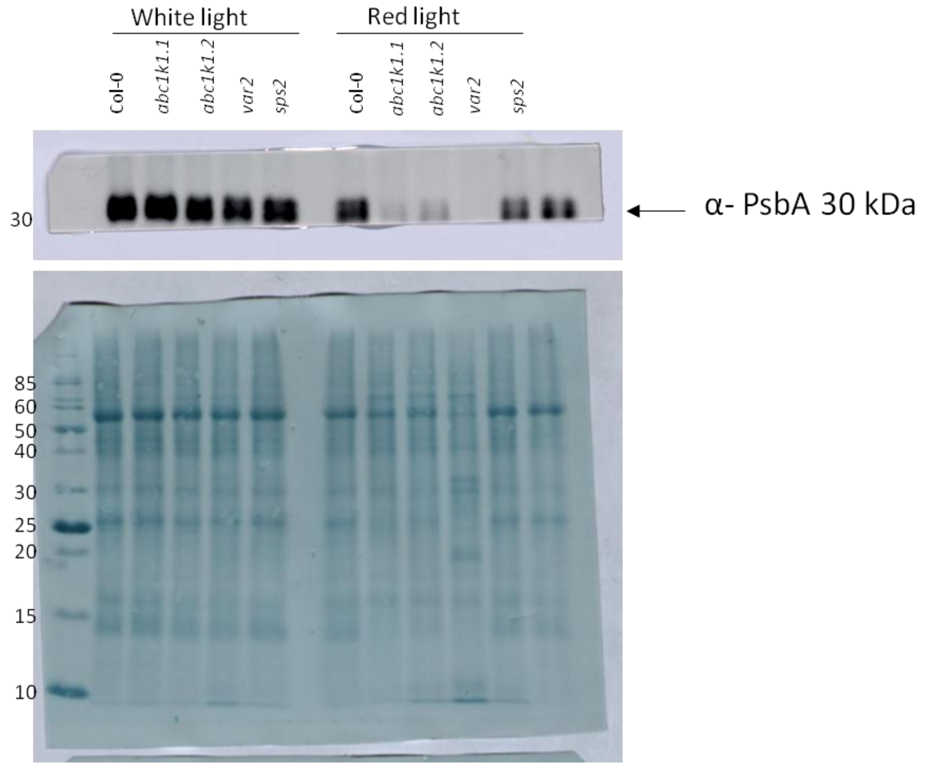

PsbA Replicate 2

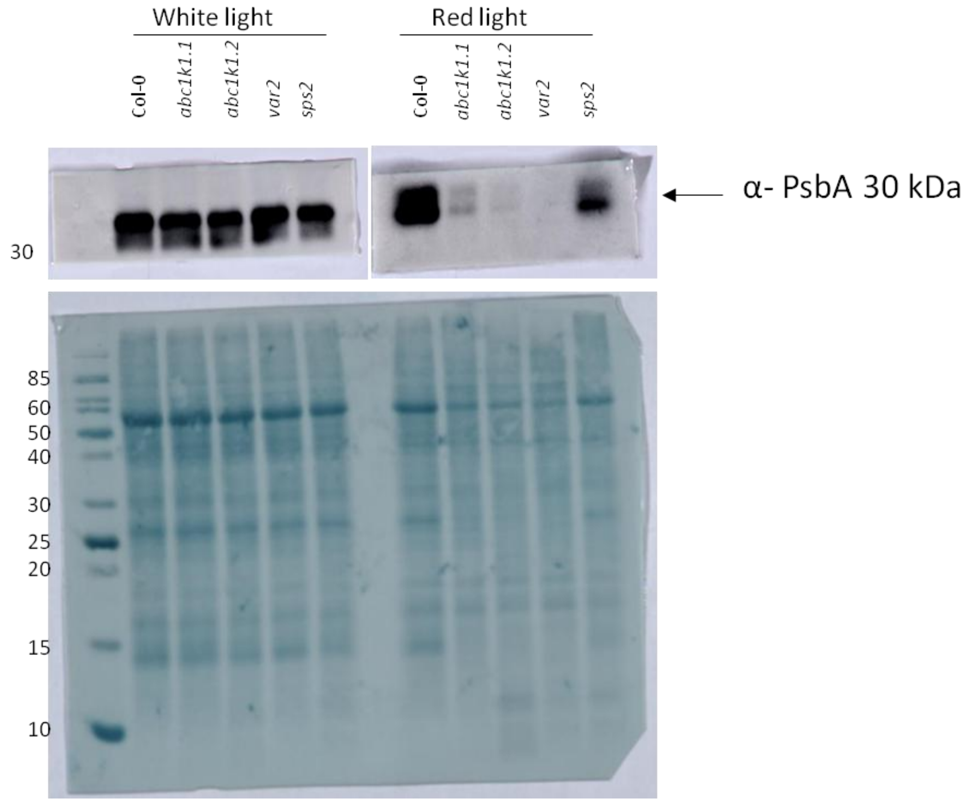

PsbA Replicate 3

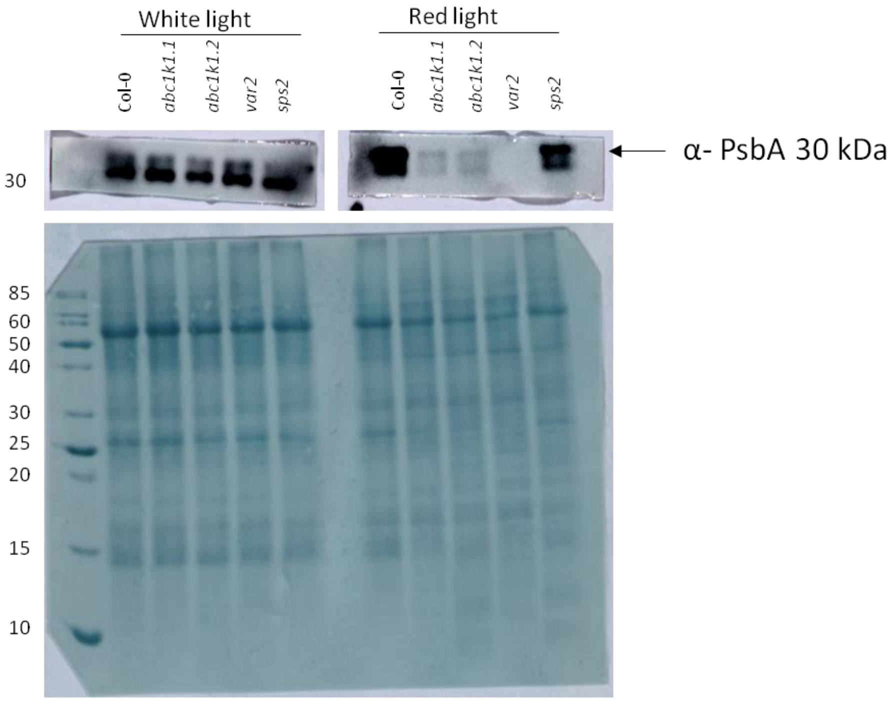

PsbA Replicate 4

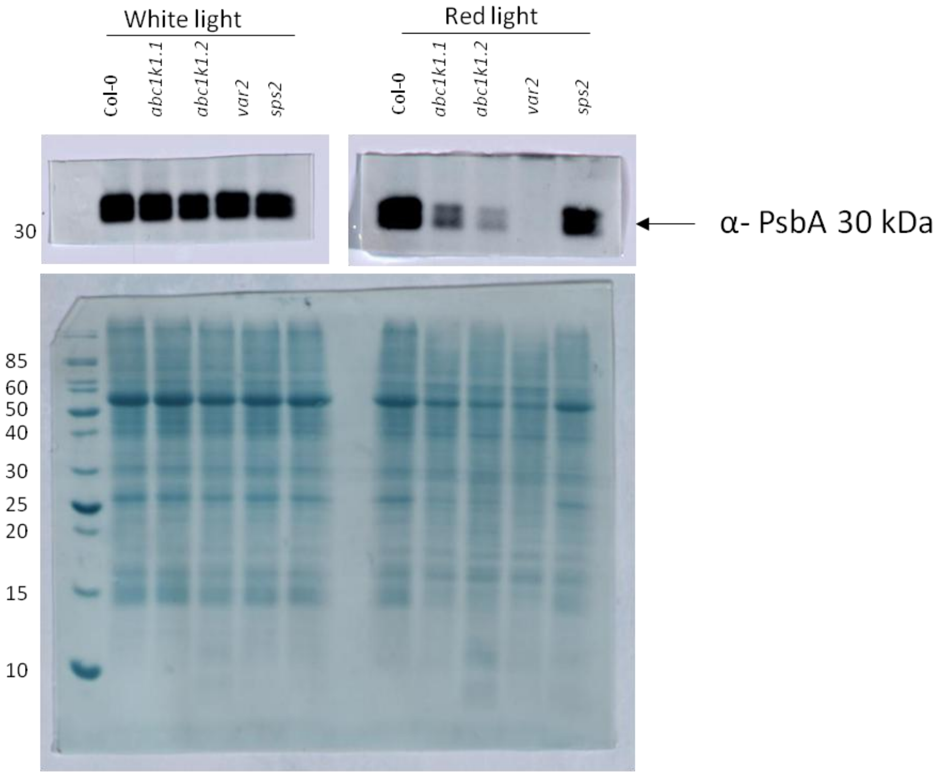

PsbB Replicate 1

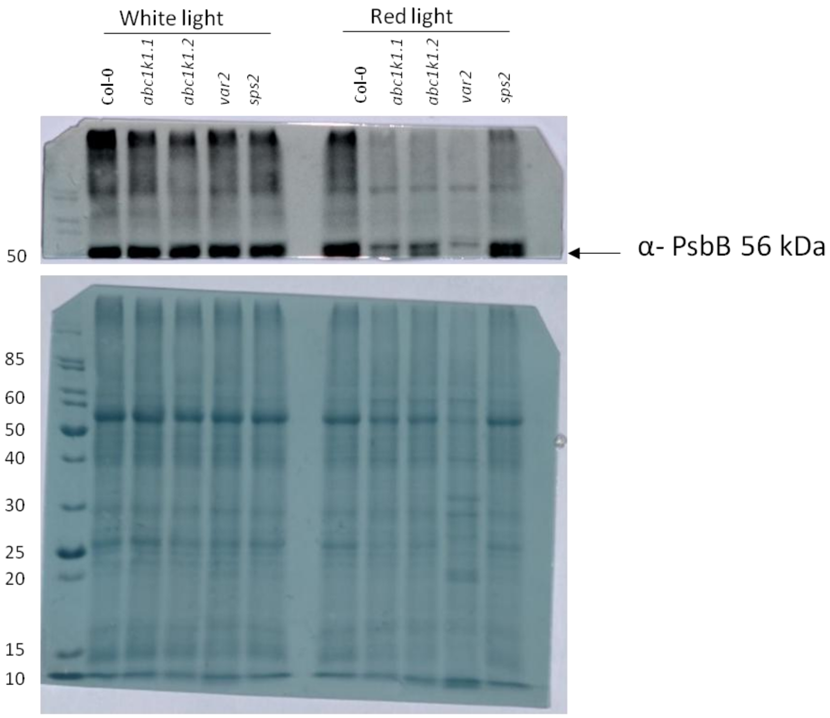

PsbB Replicate 2

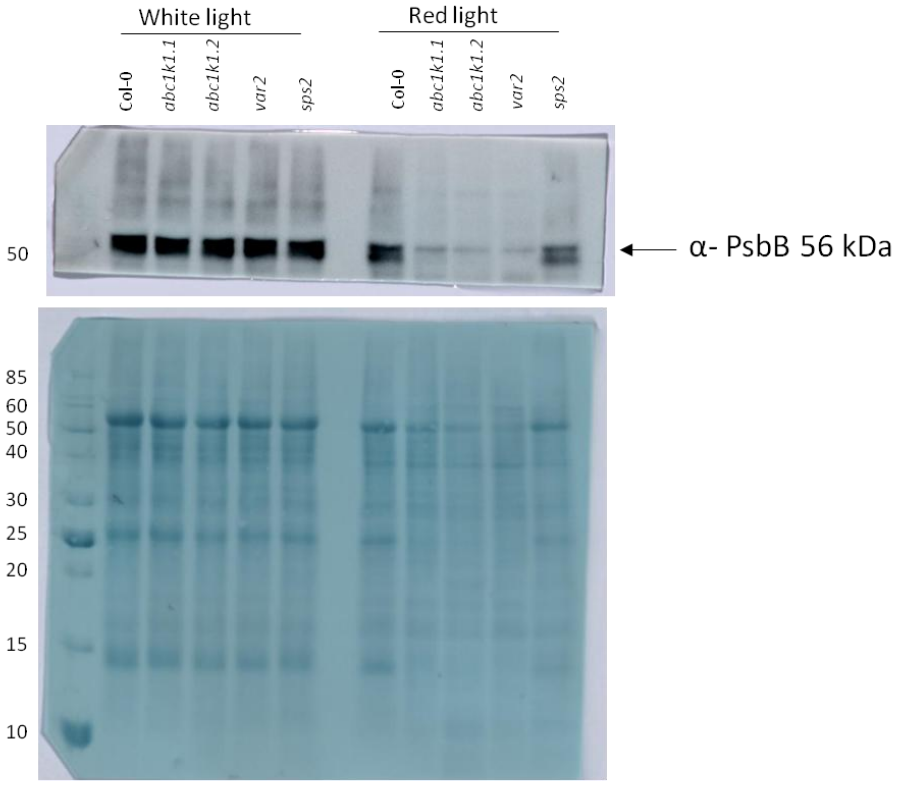

PsbB Replicate 3

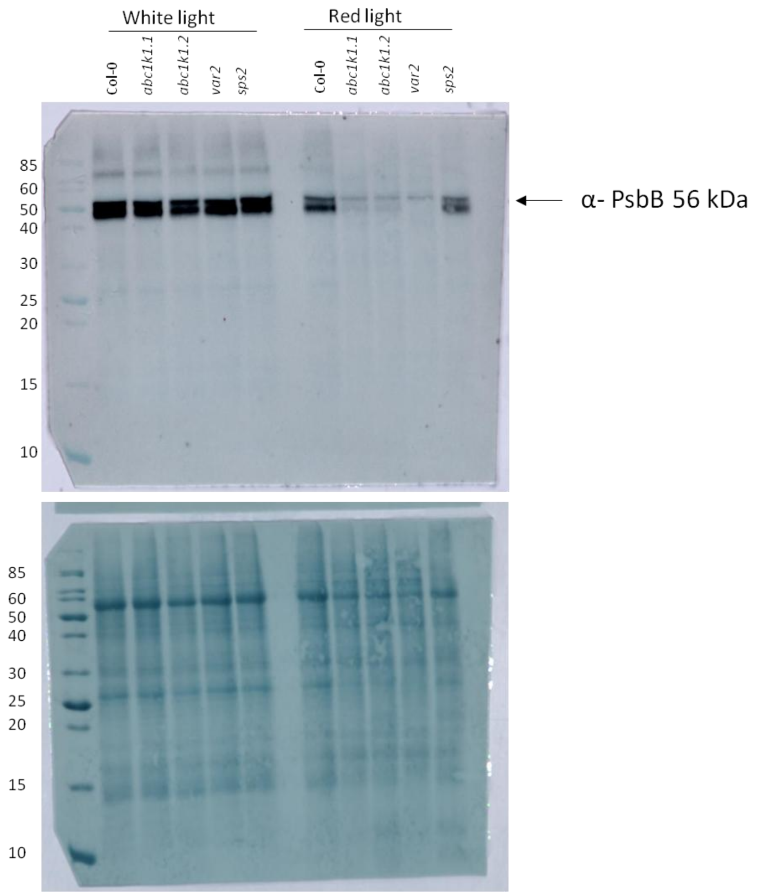

PsbB Replicate 4

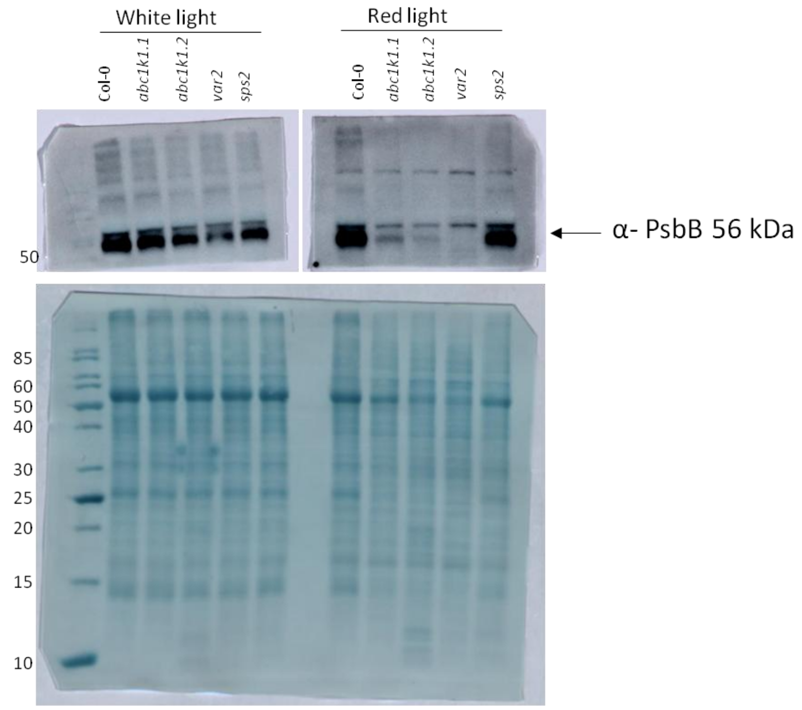

Psbo1 Replicate 1

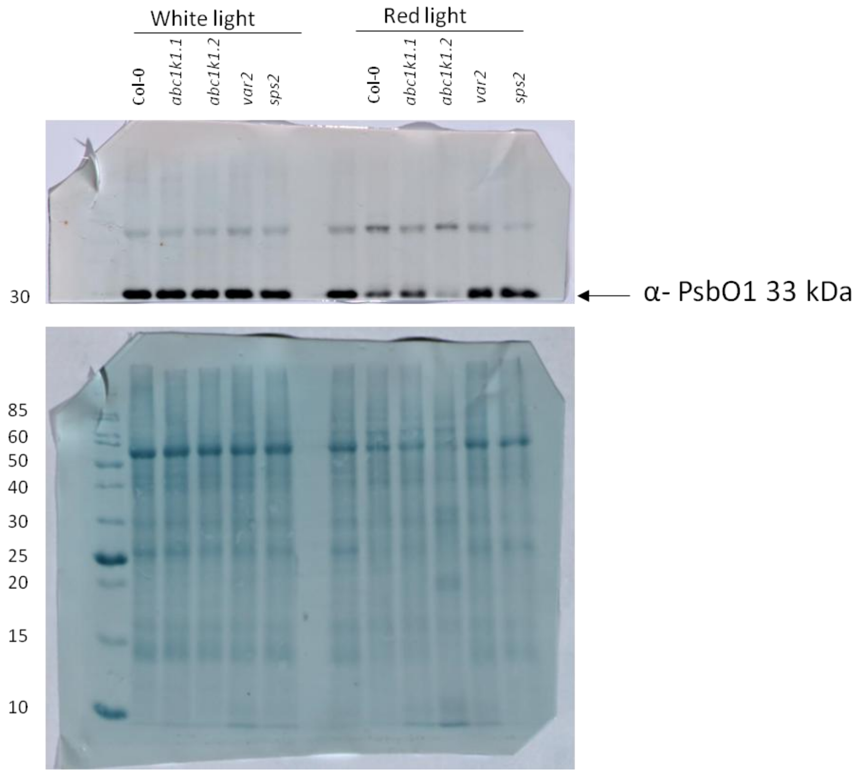

PsbO1 Replicate 2

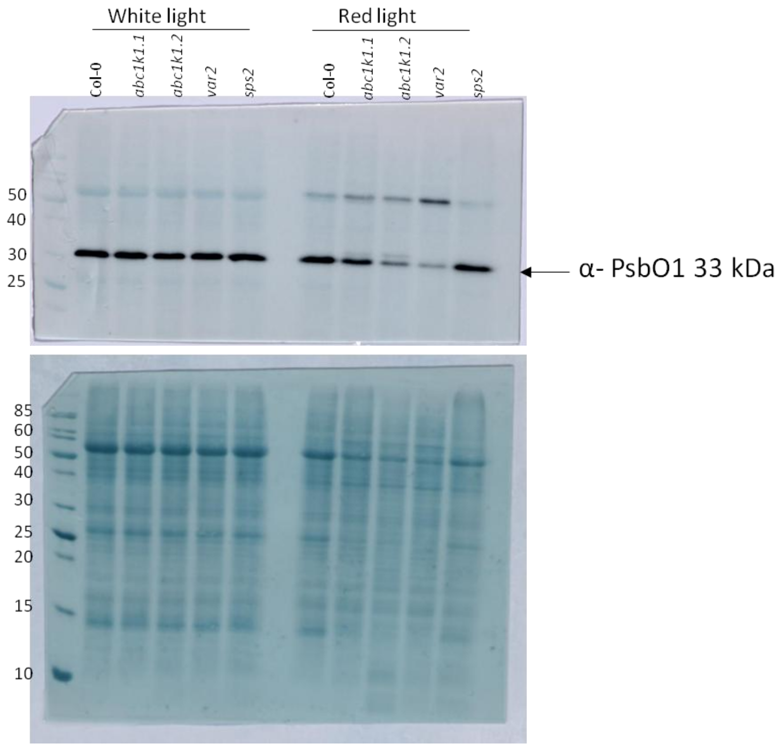

PsbO1 Replicate 3

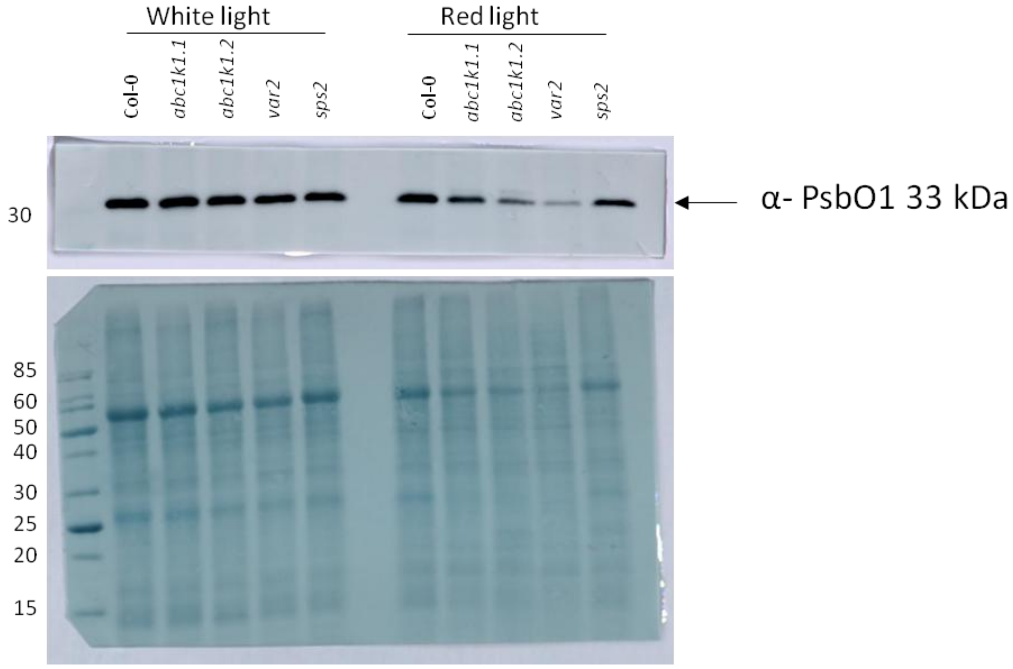

PsbO1 Replicate 4

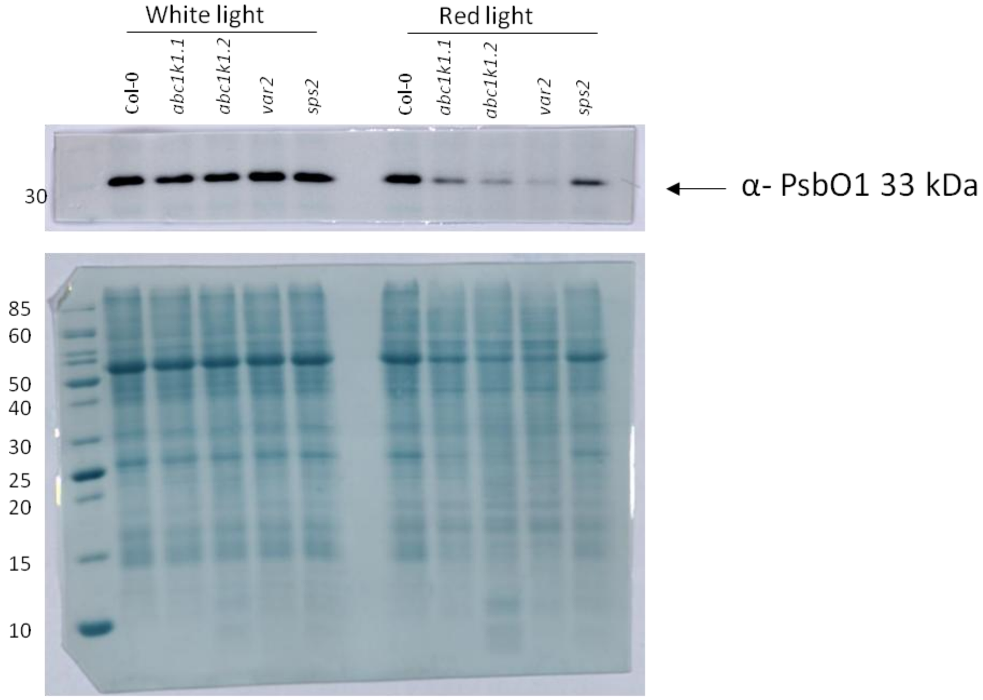

PsbQ Replicate 1

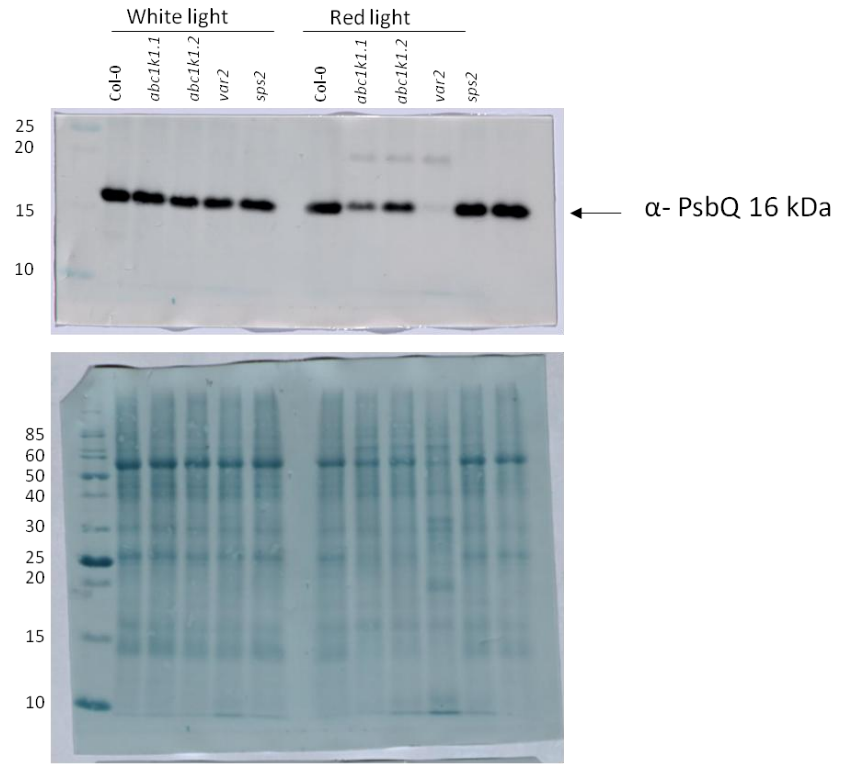

PsbQ Replicate 2

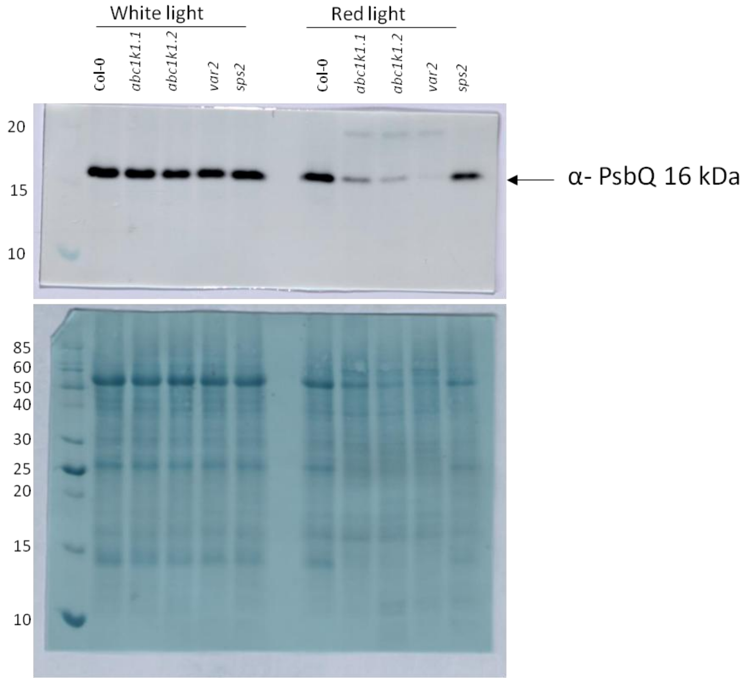

PsbQ Replicate 3

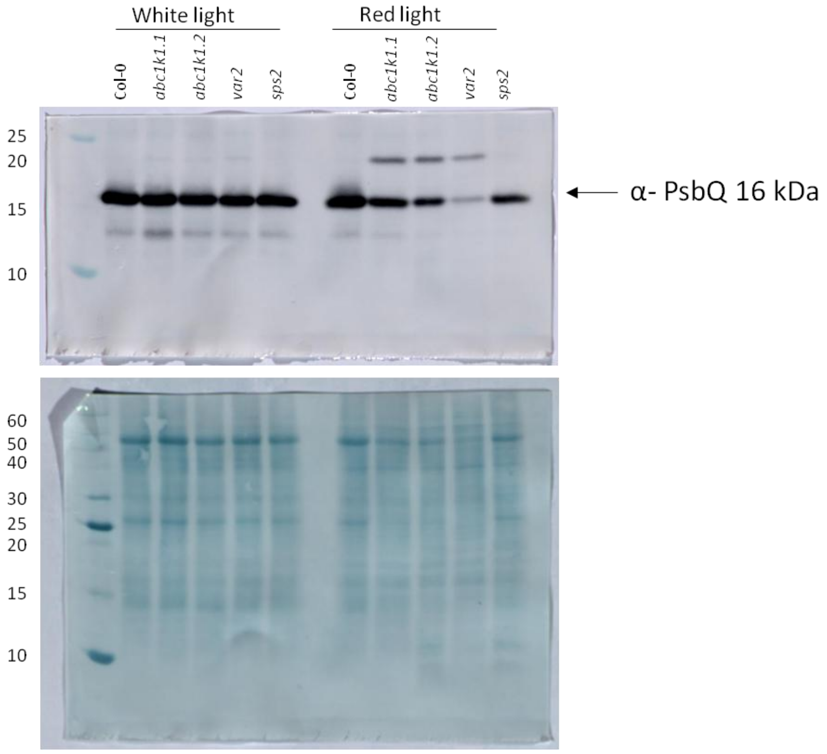

PsbQ Replicate 4

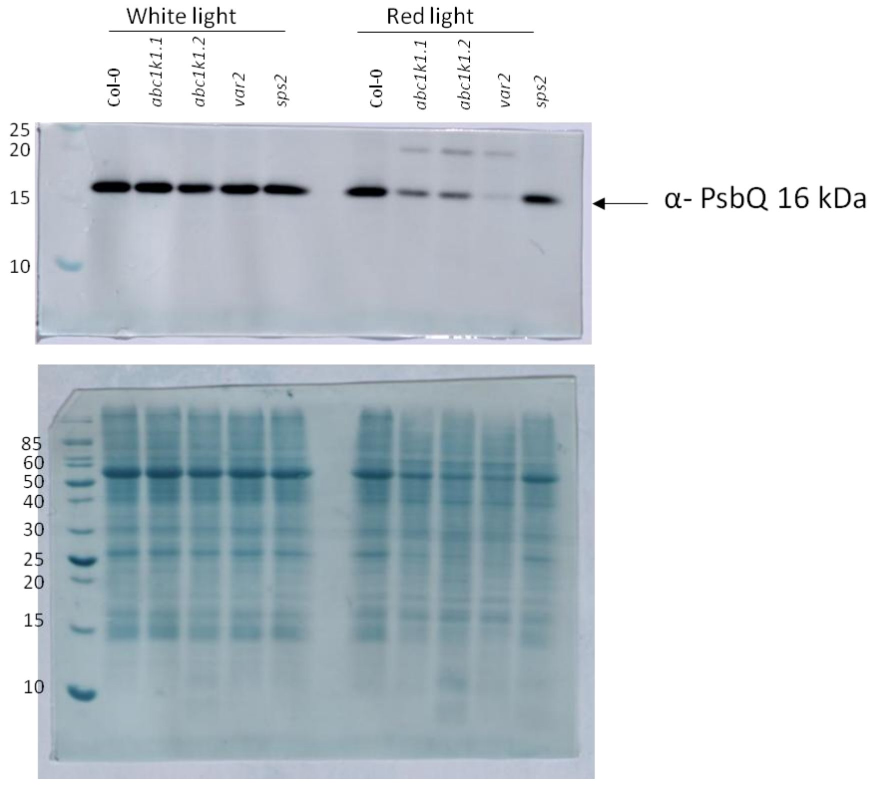

PsbP Replicate 1

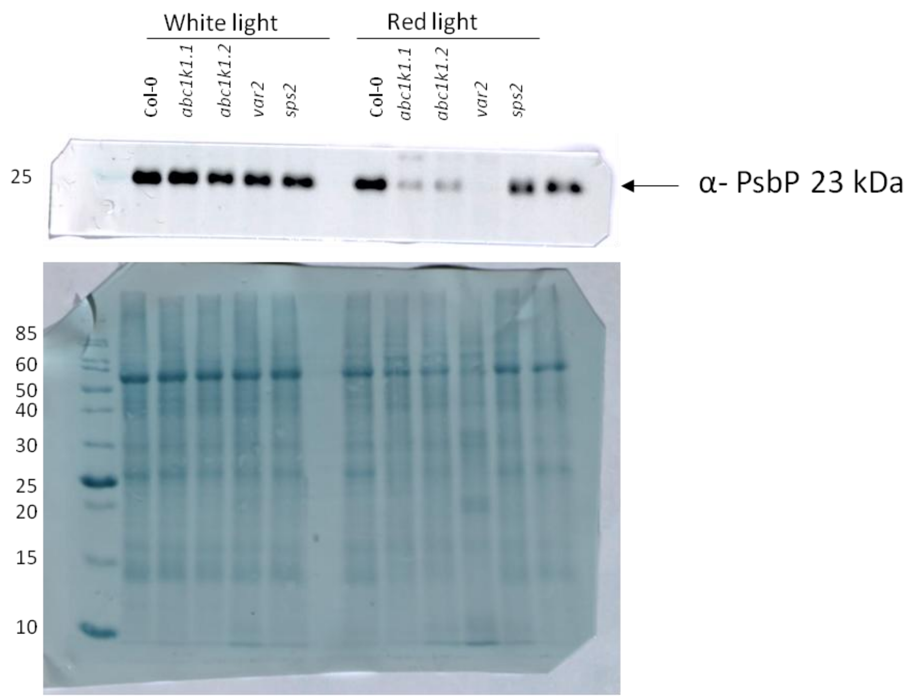

PsbP Replicate 2

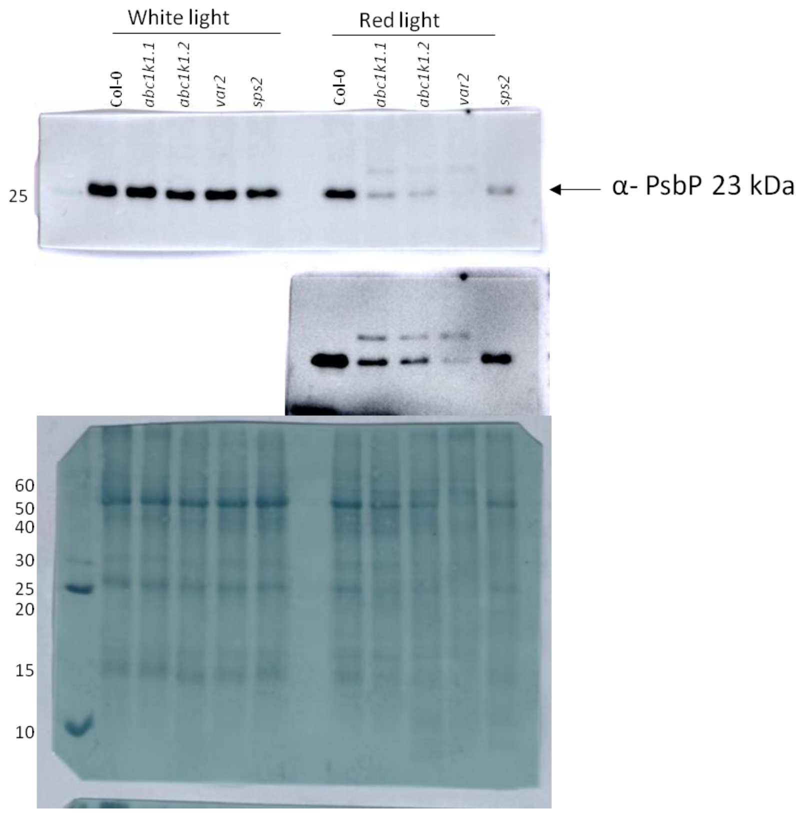

PsbP Replicate 3

*

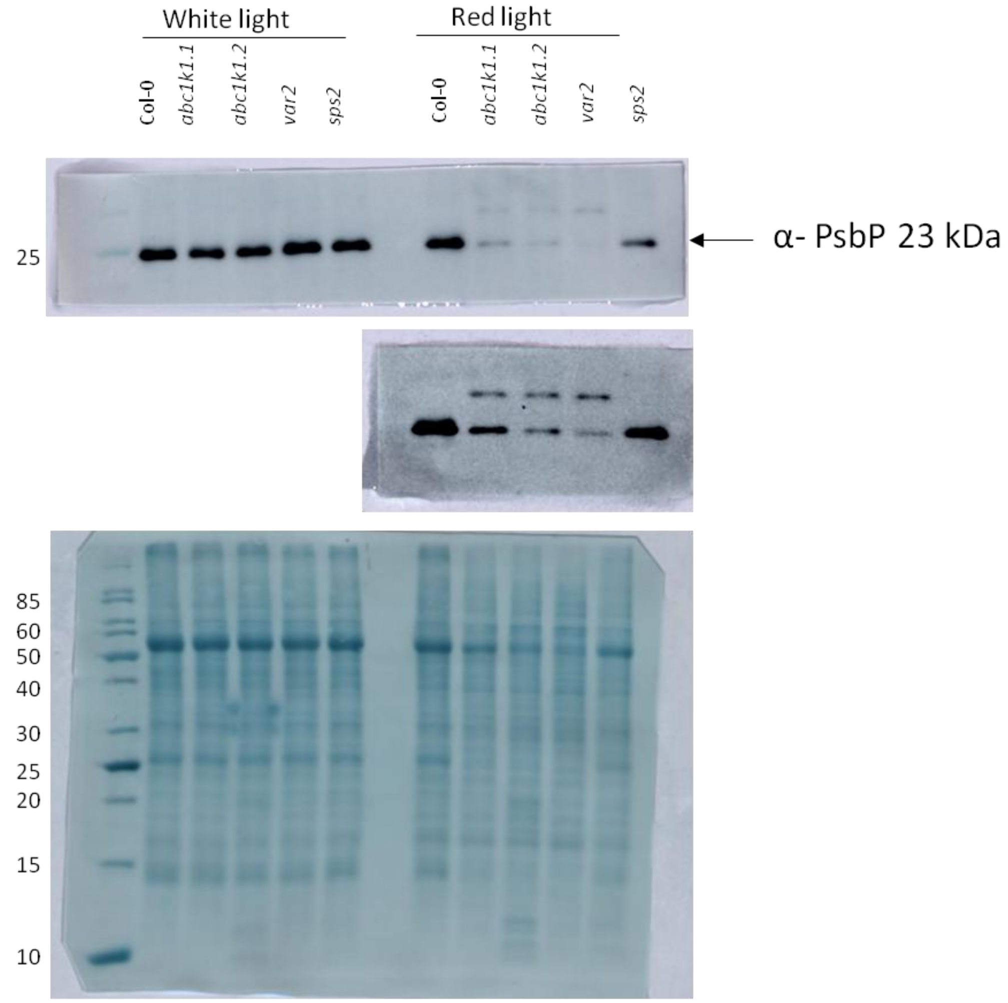

PsaD Replicate 1

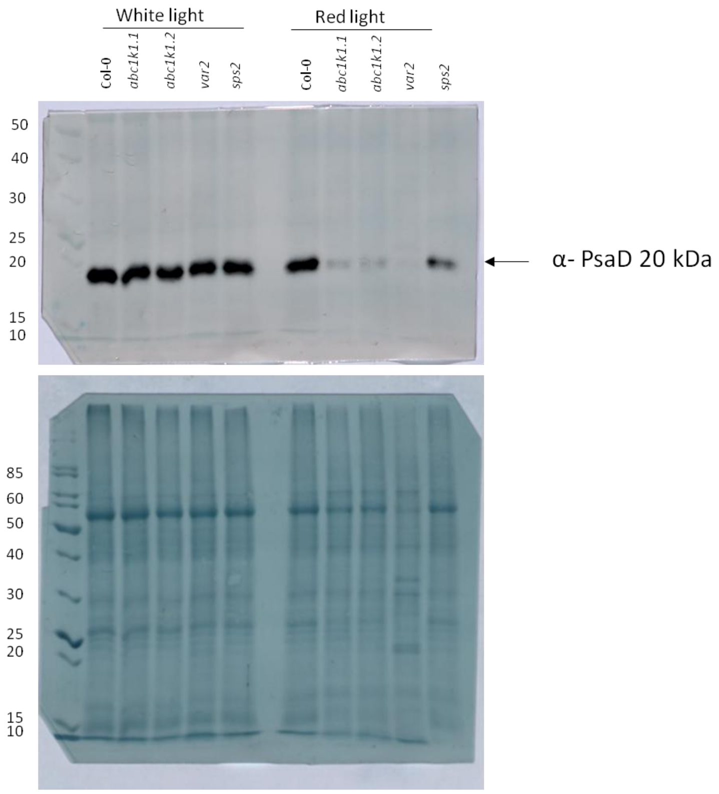

PsaD Replicate 2

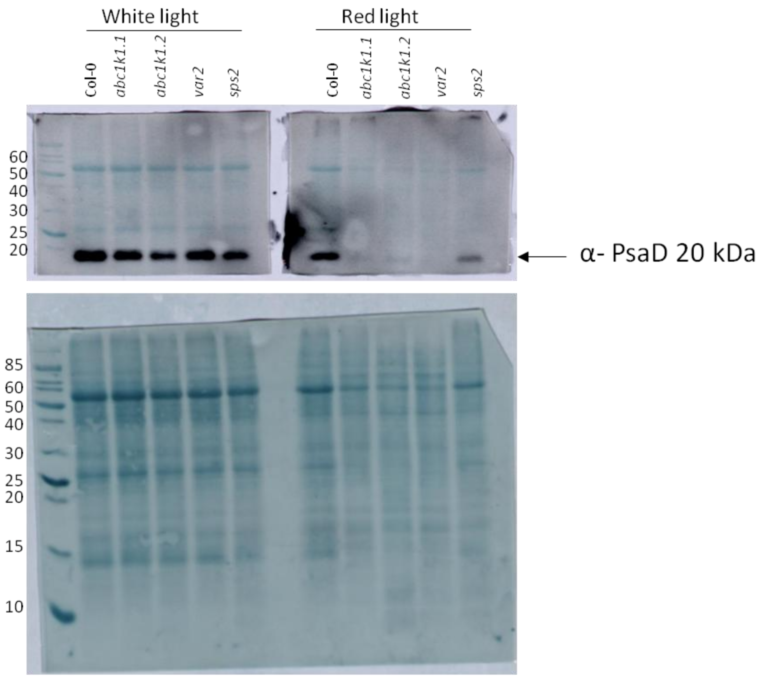

PsaD Replicate 3

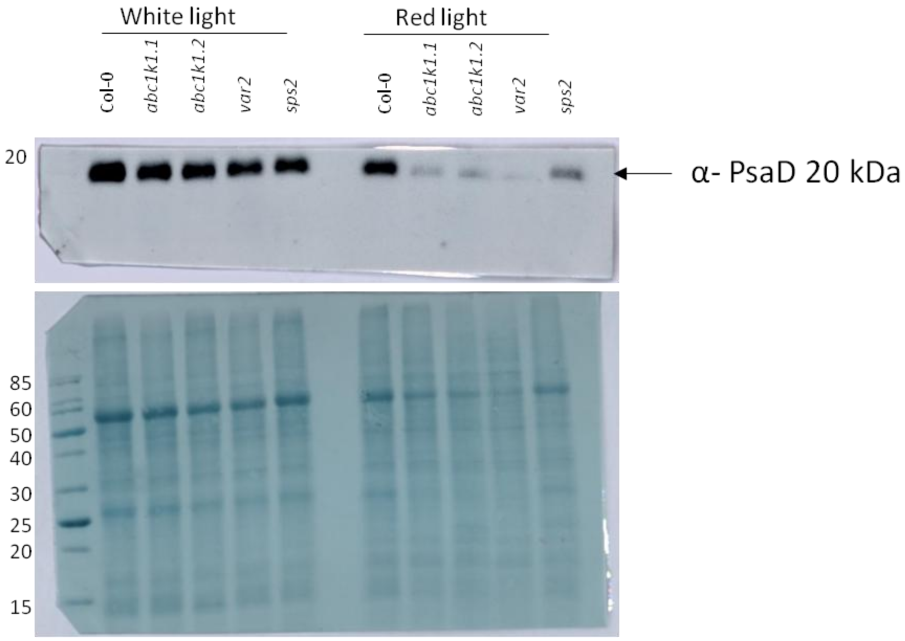

PsaD Replicate 4

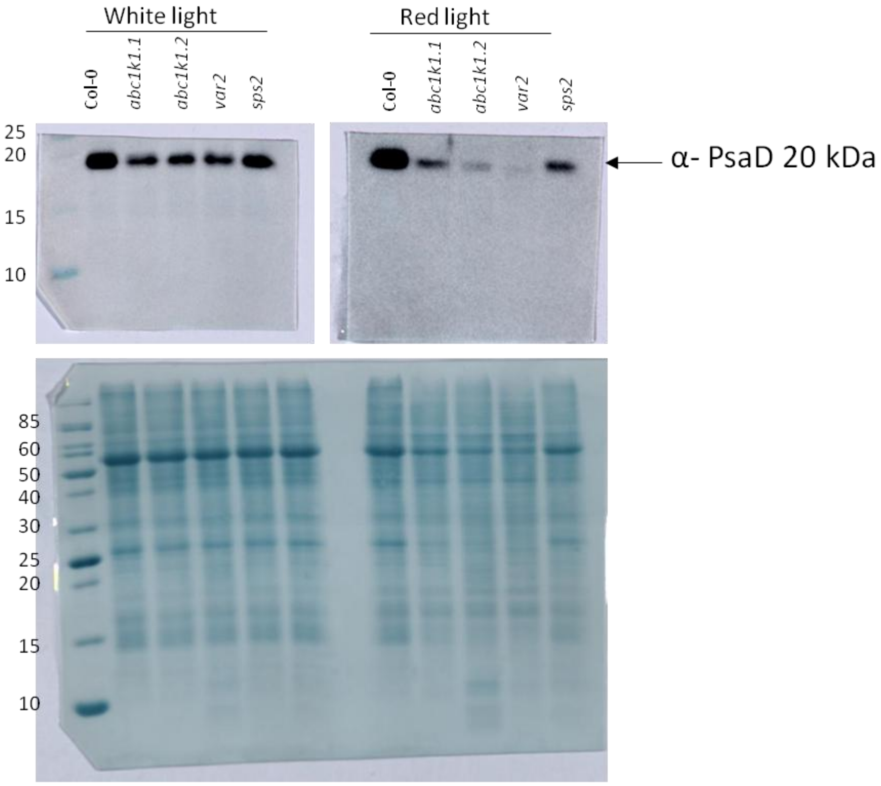

PsaN Replicate 1

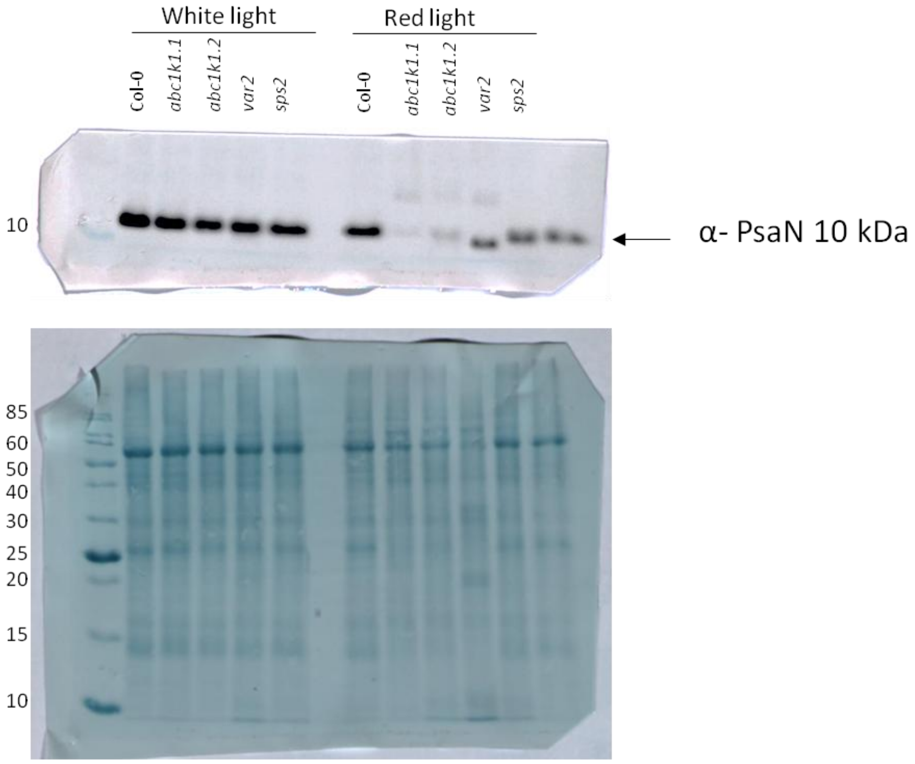

PsaN Replicate 2

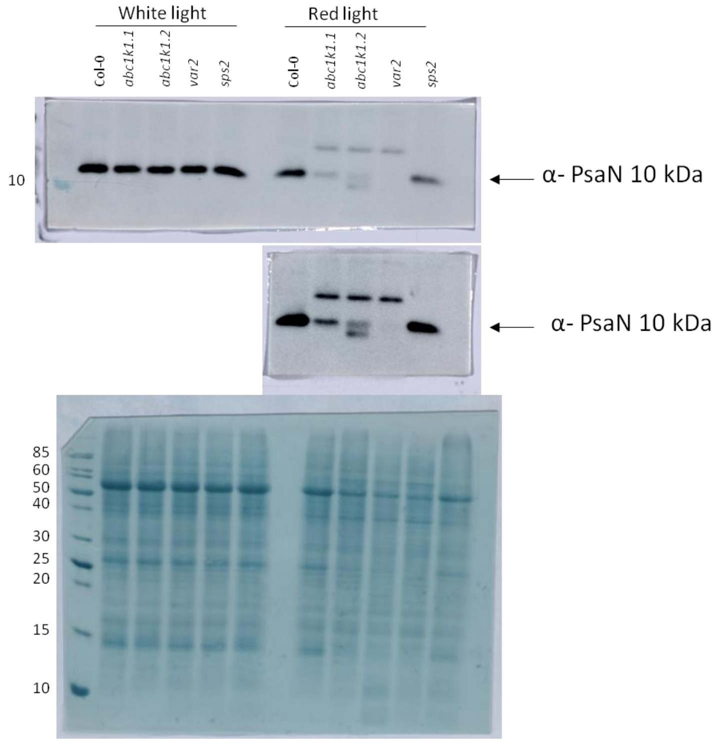

PsaN Replicate 3

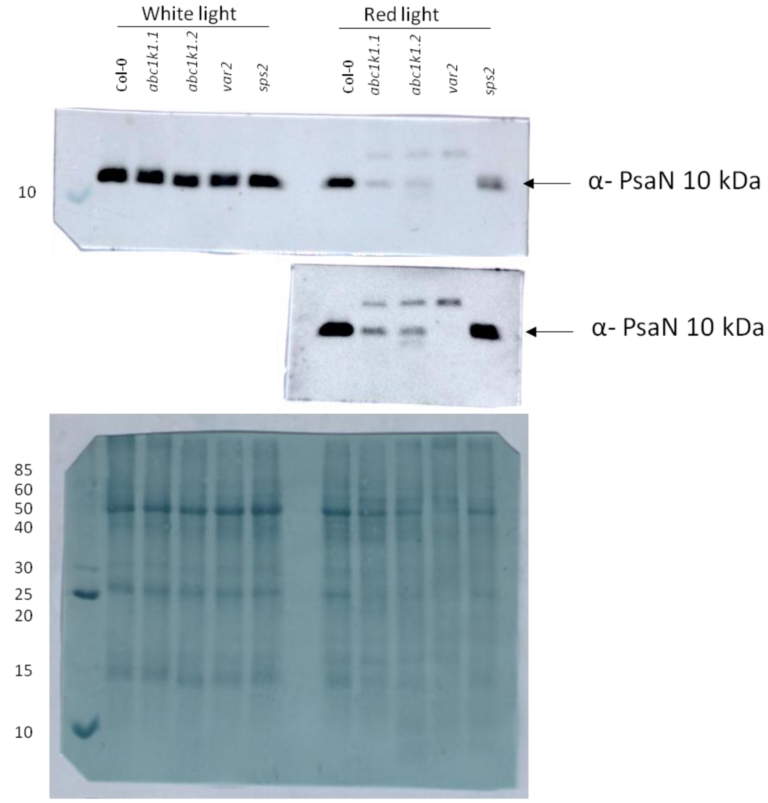

PsaN Replicate 4

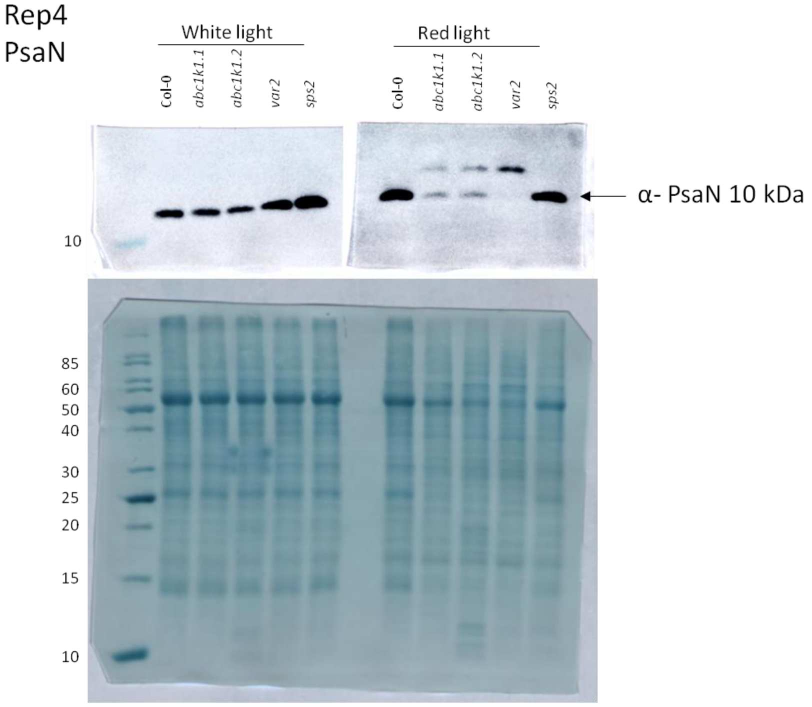

PetB Replicate 1

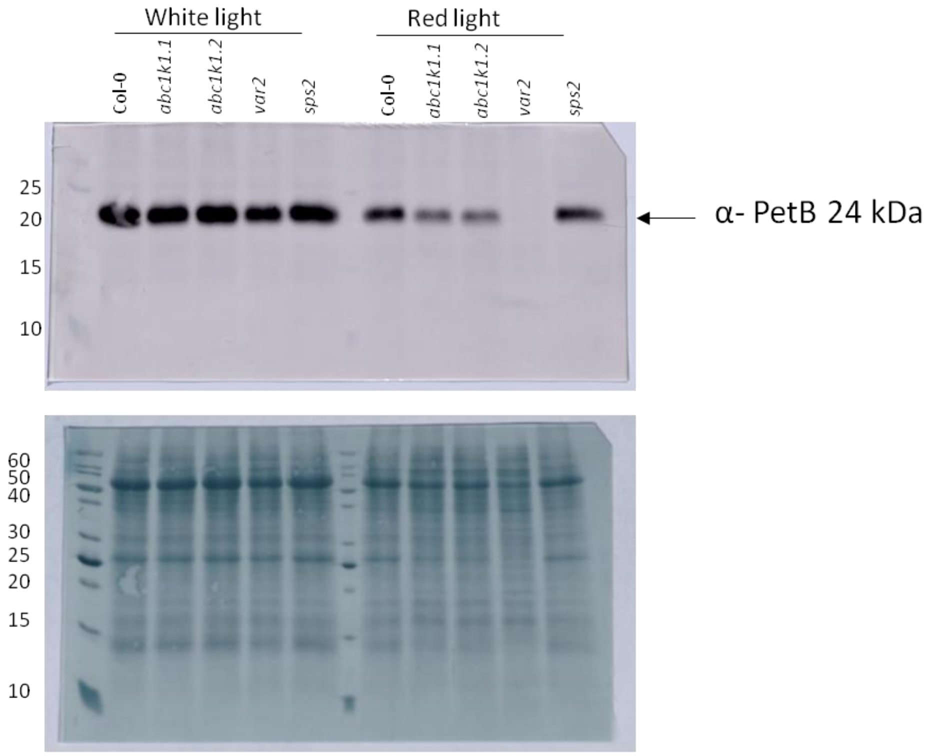

PetB Replicate 2

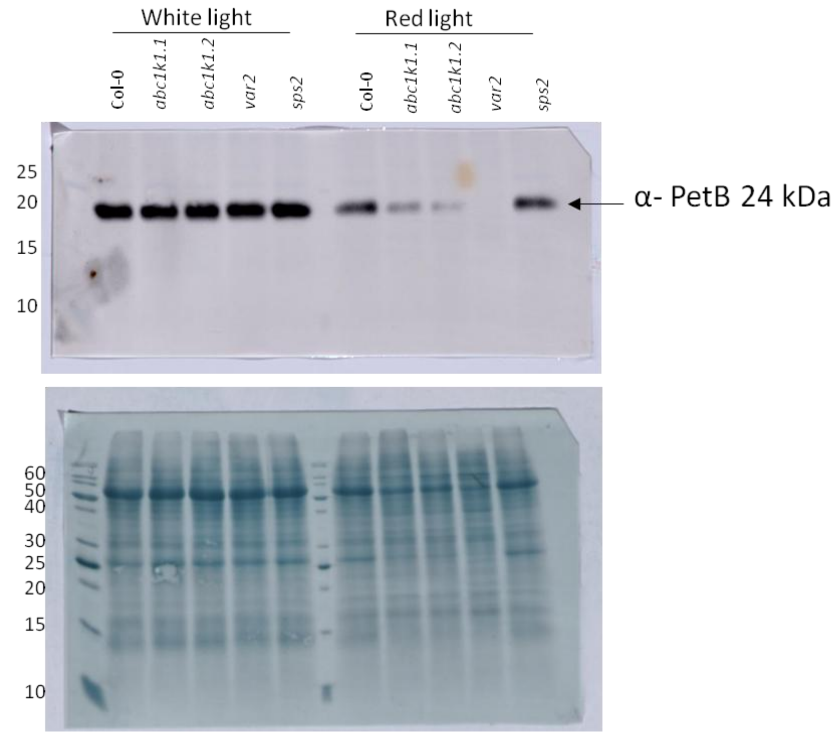

PetC Replicate 1

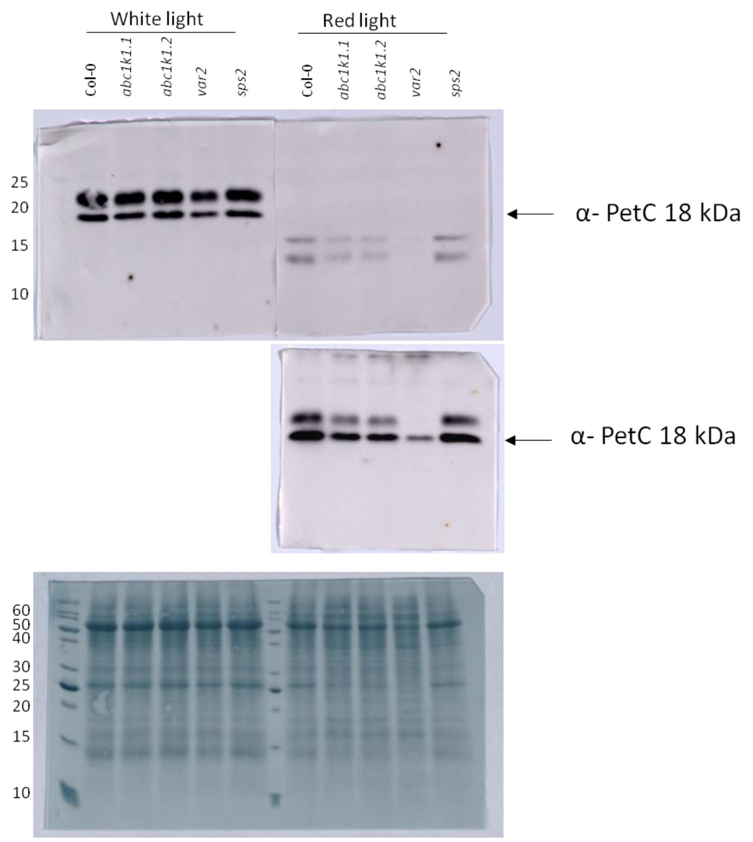

PetC Replicate 2

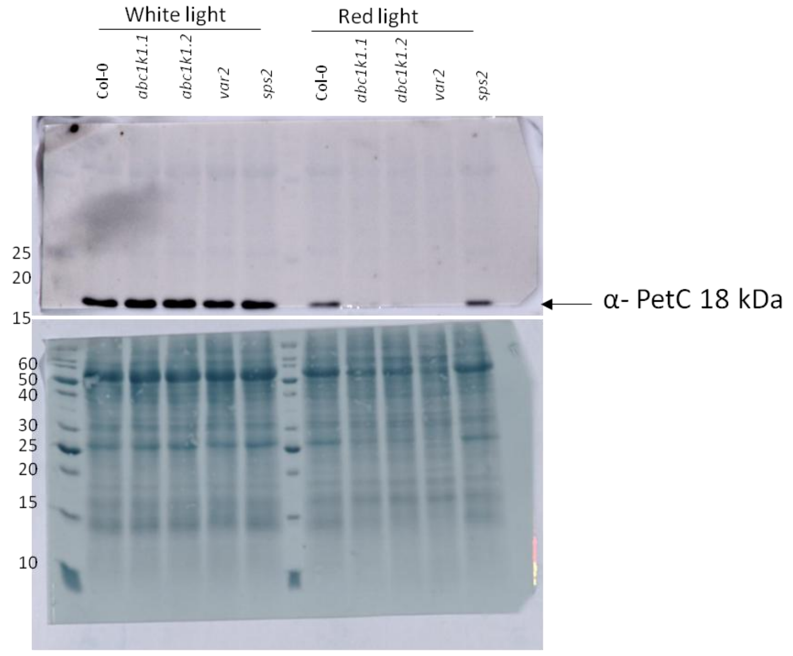

Plastocyanine PC Replicate 1

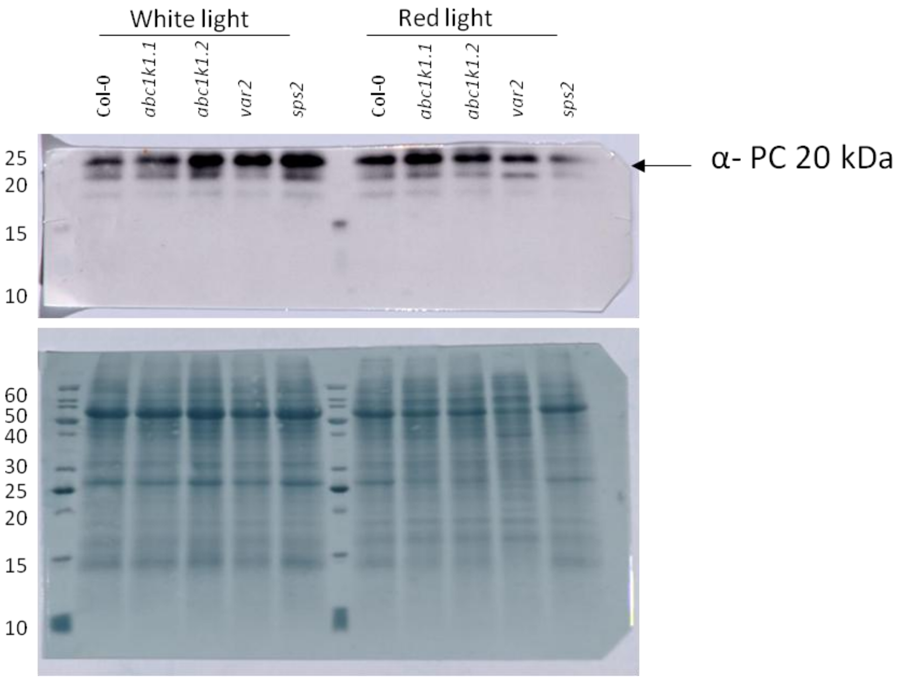

Plastocyanine PC Replicate 2

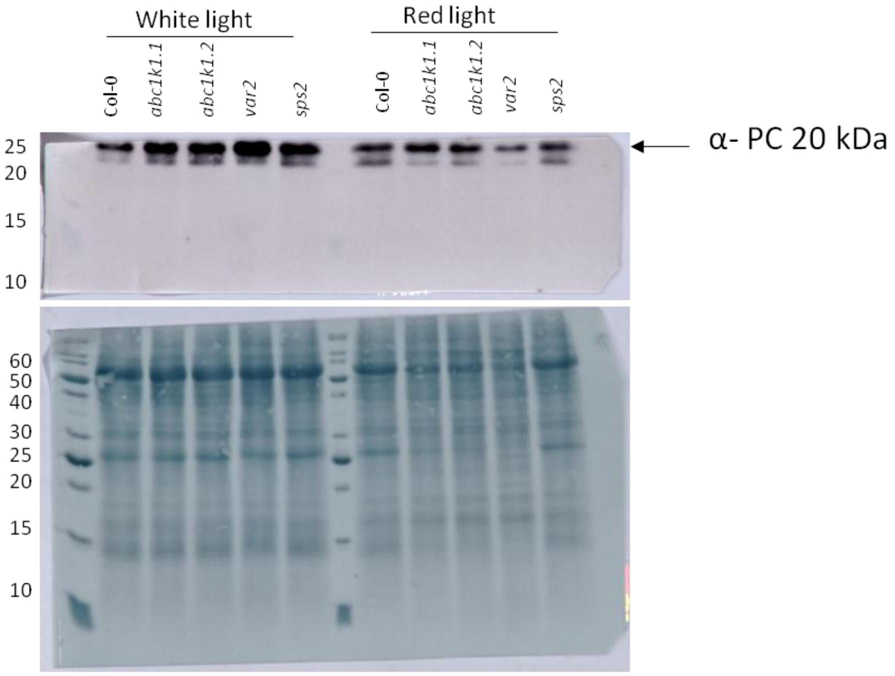

Lhcb1 Replicate 1

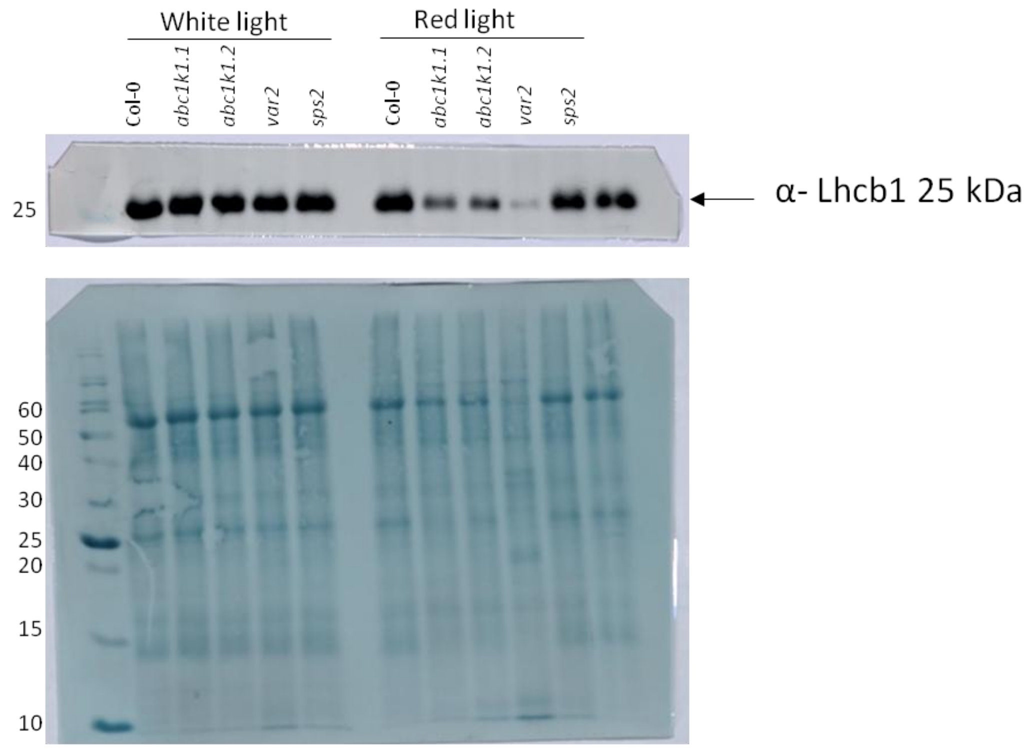

Lhcb1 Replicate 2

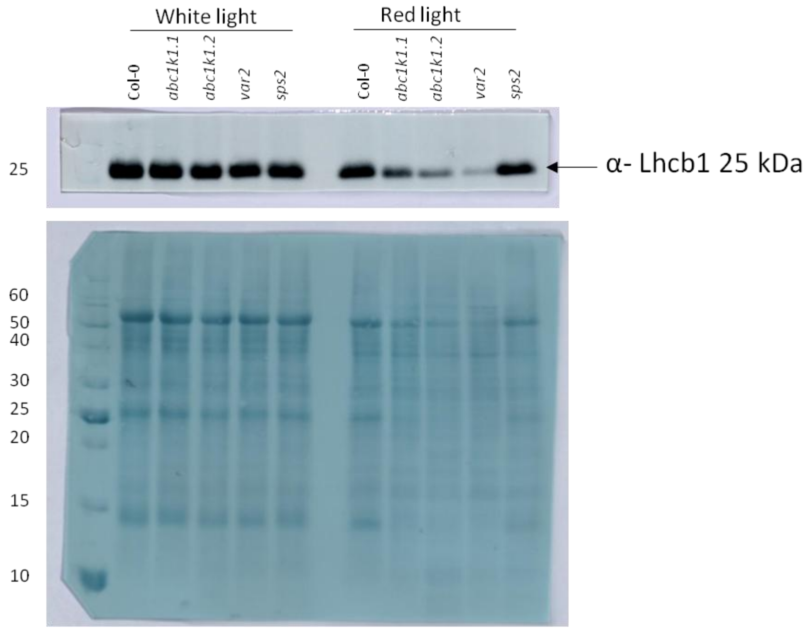

Lhcb1 Replicate 3

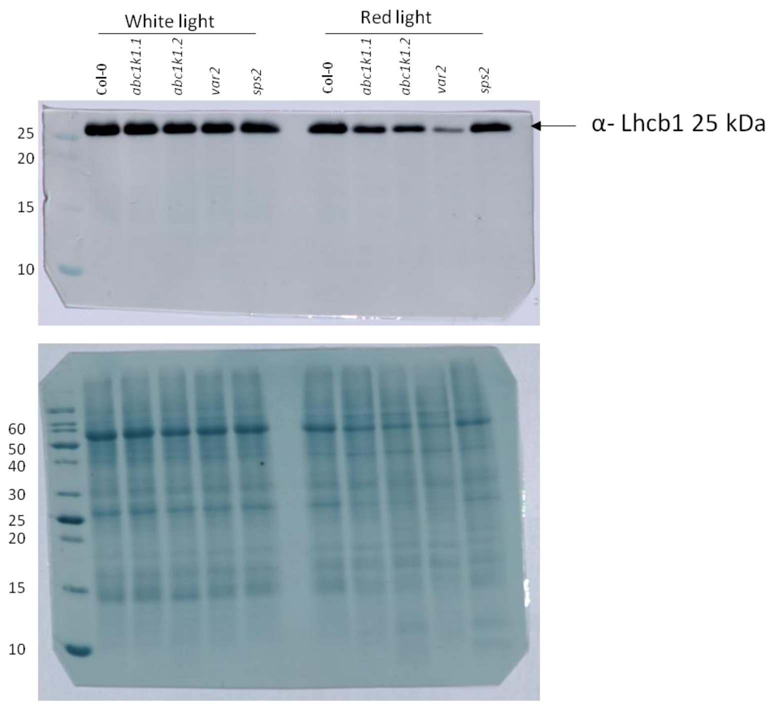

Lhcb1 Replicate 4

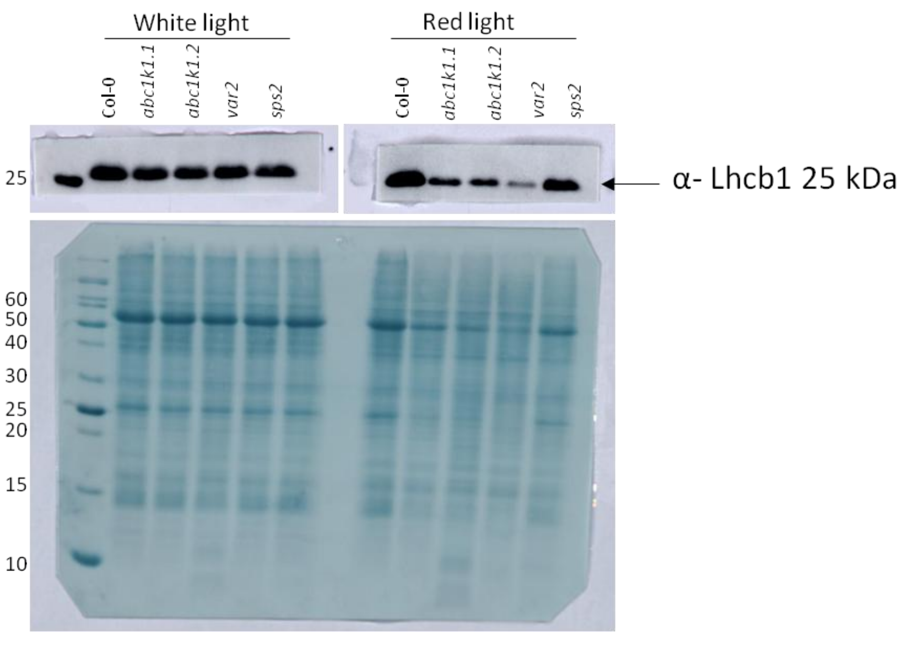

Lhca1 Replicate 1

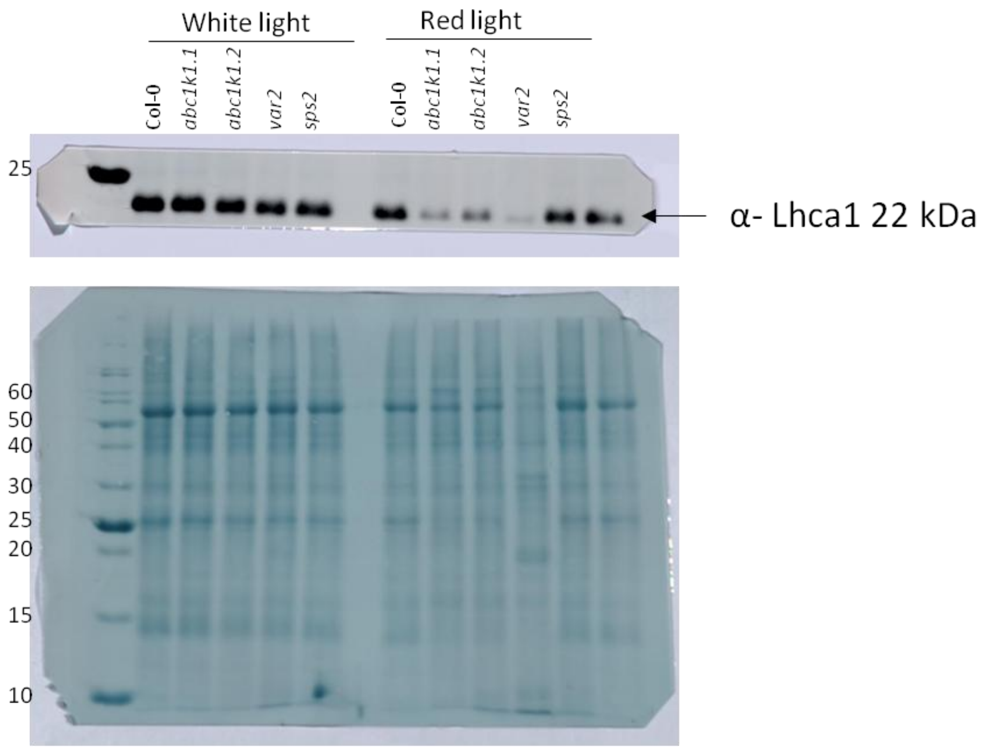

Lhca1 Replicate 2

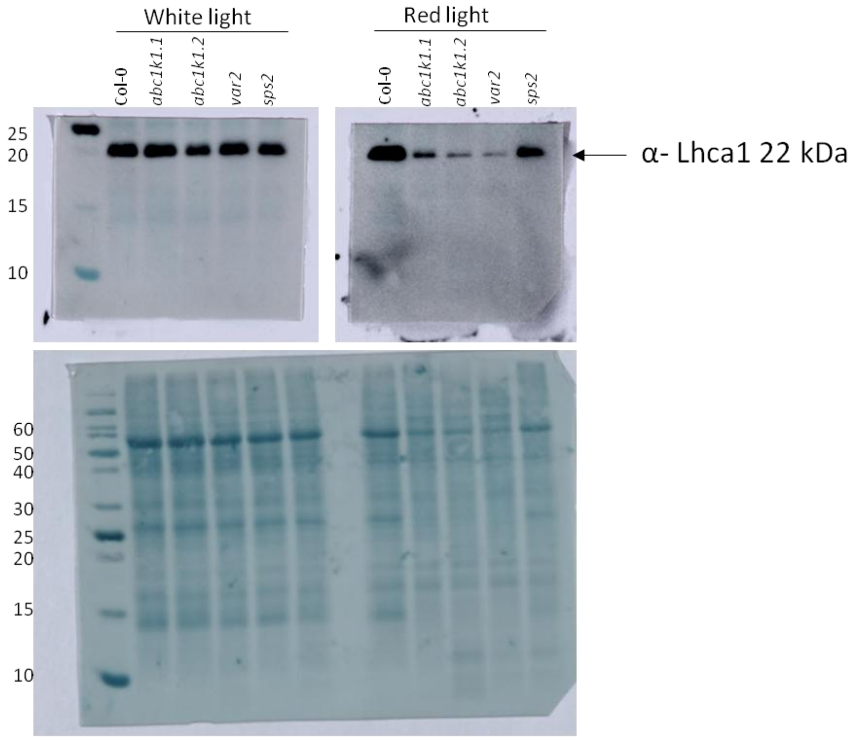

Lhca1 Replicate 3

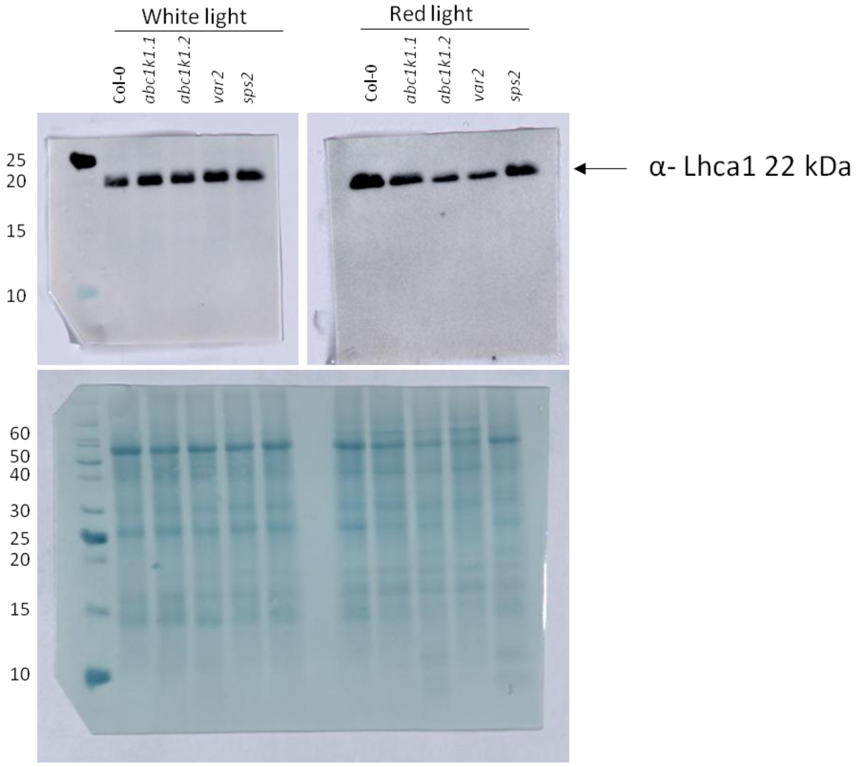

Lhca1 Replicate 4

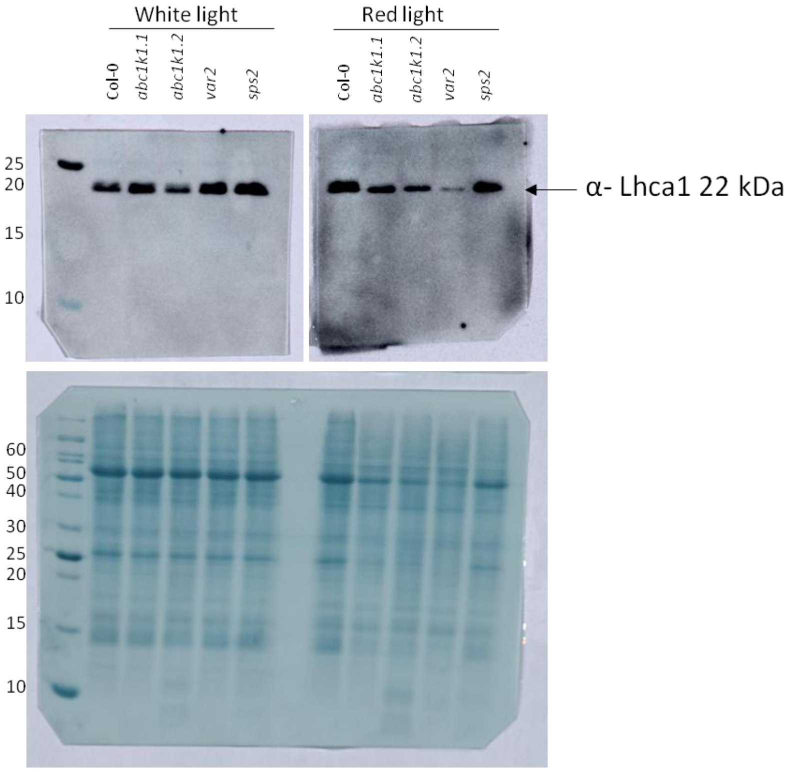

Toc 75 Replicate 1

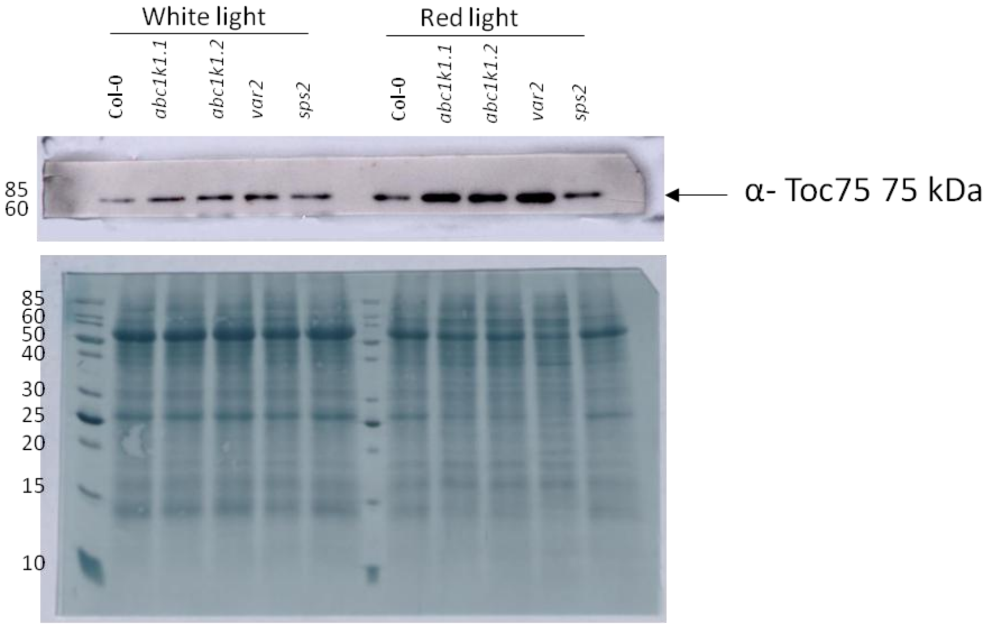

Toc75 Replicate 2

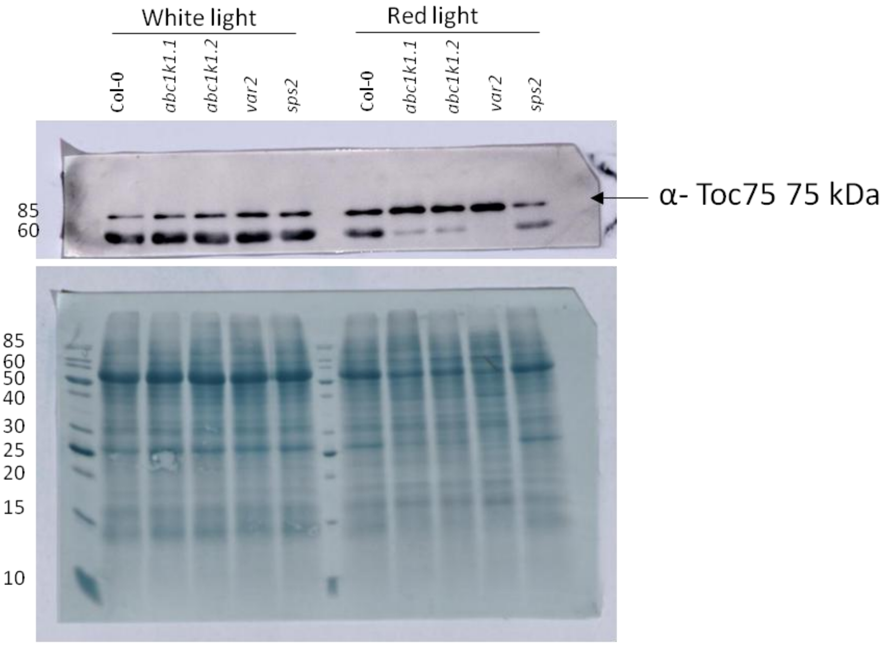

Lhcb1-P Replicate 1

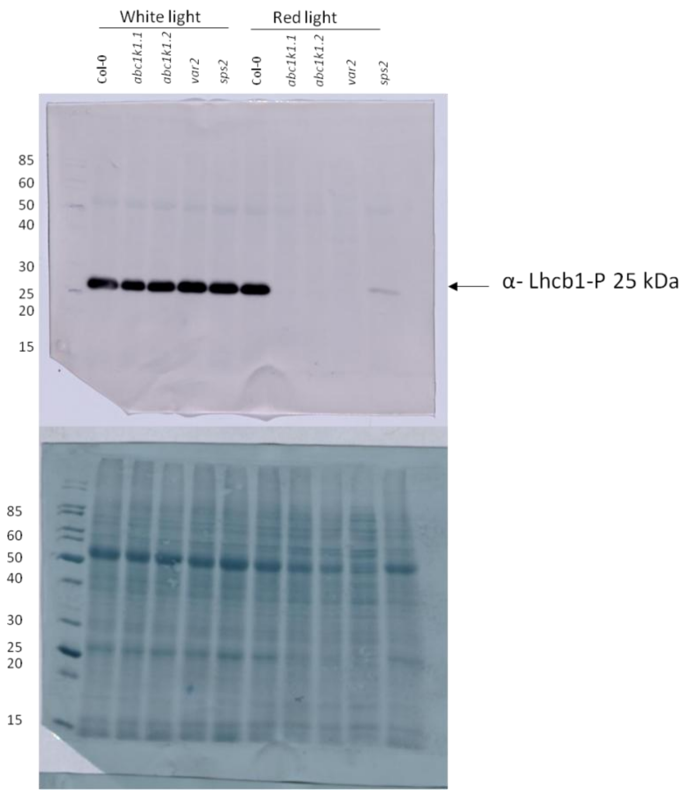

Lhcb1-P Replicate 2

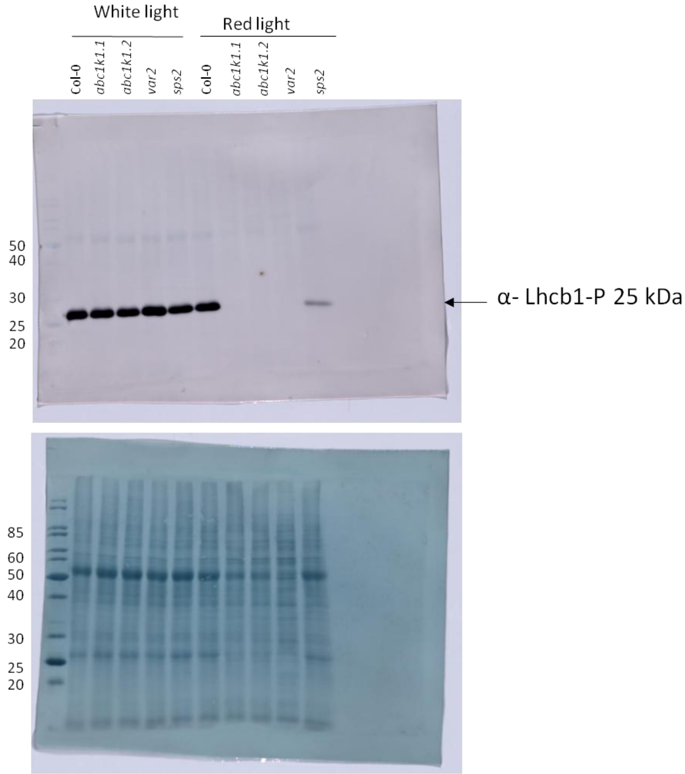

Lhcb1-P Replicate 3

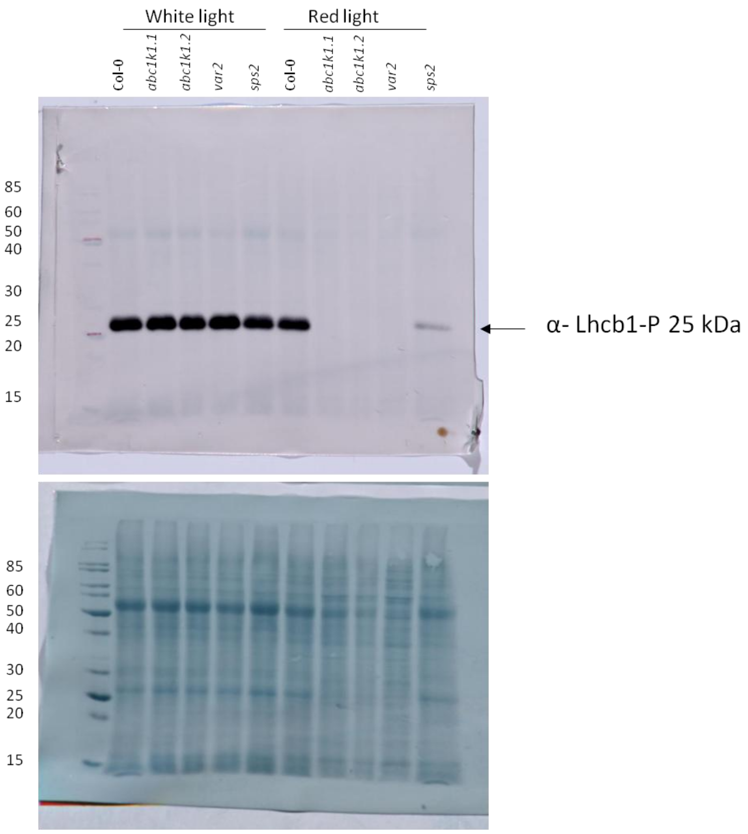

PsbA-P Replicate 1

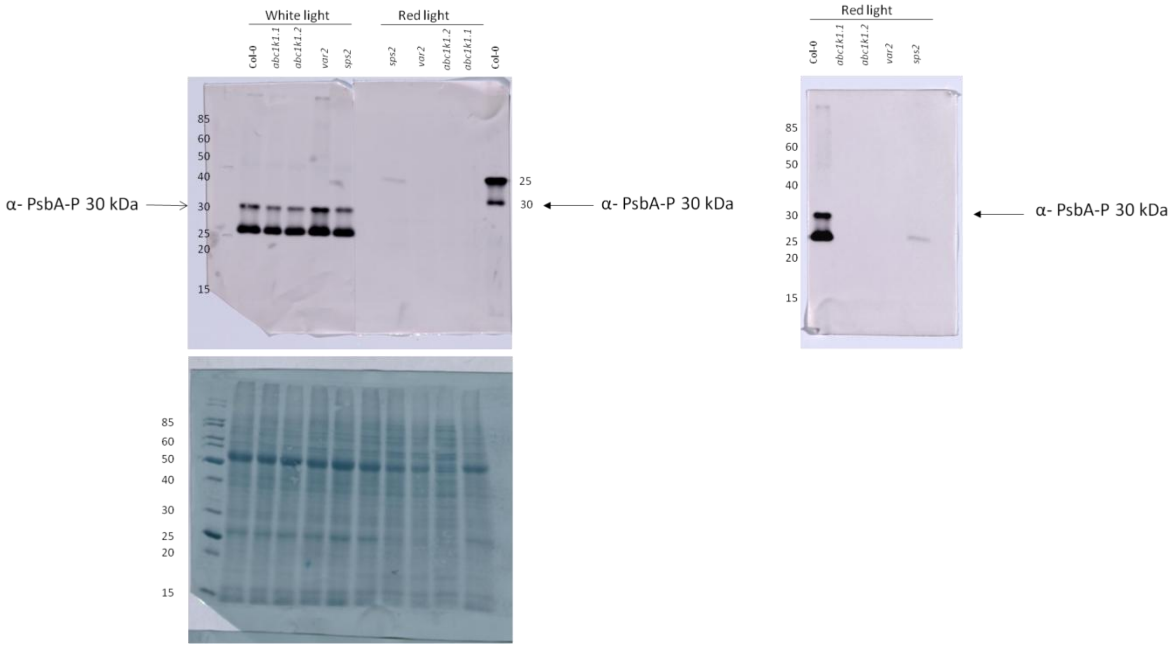

PsbA-P Replicate 2

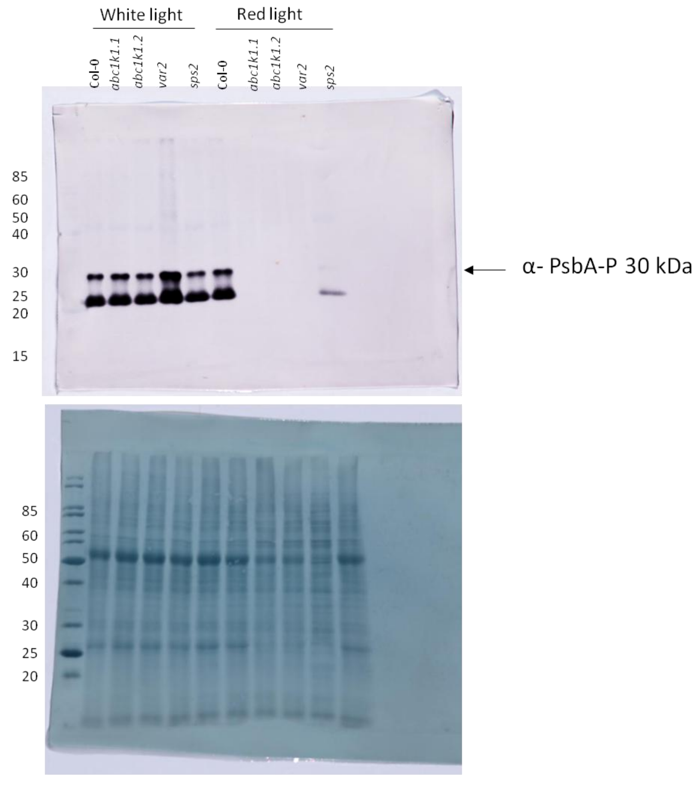

PsbA-P Replicate 3

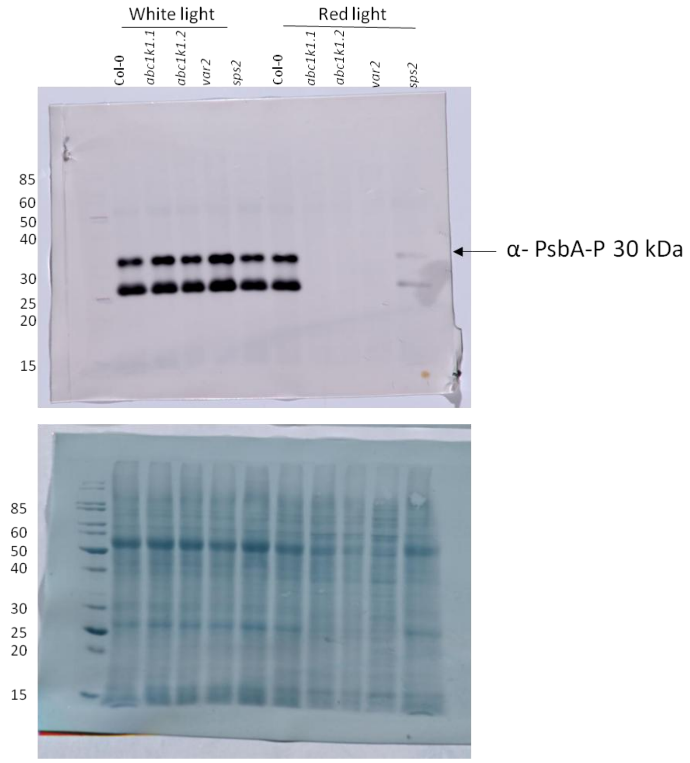

Supplementary fig. 8c

PsbP Replicate 1

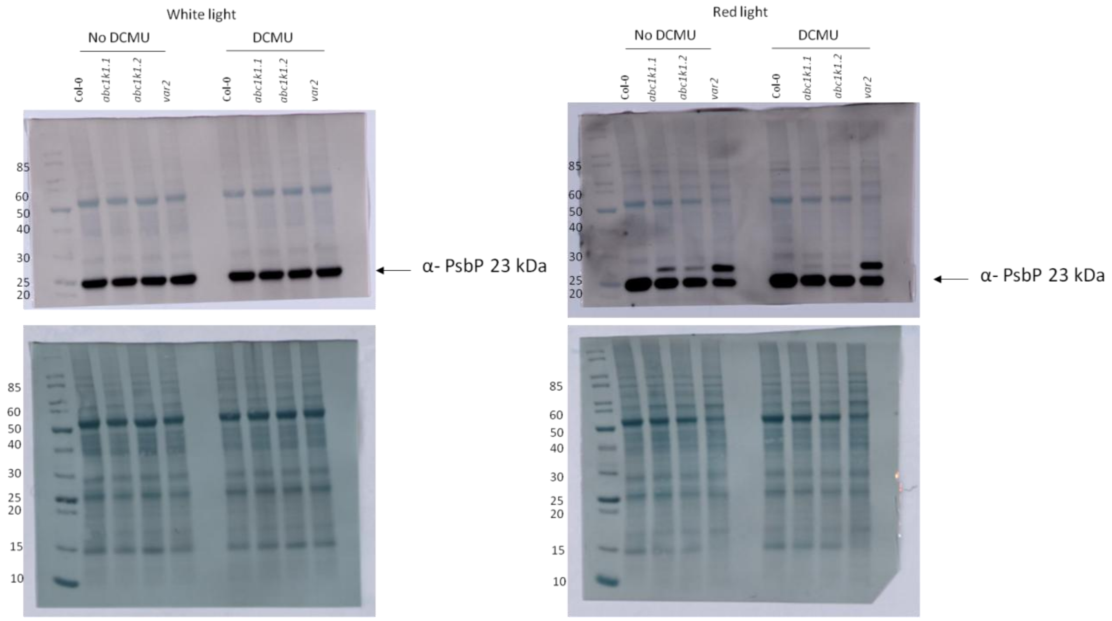

PsbP Replicate 2

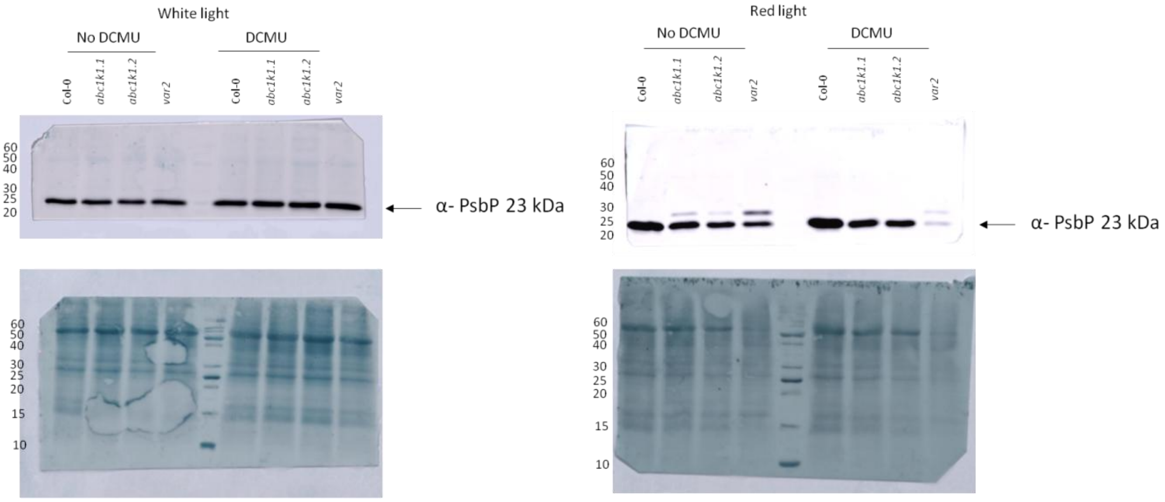

PsbQ Replicate 1

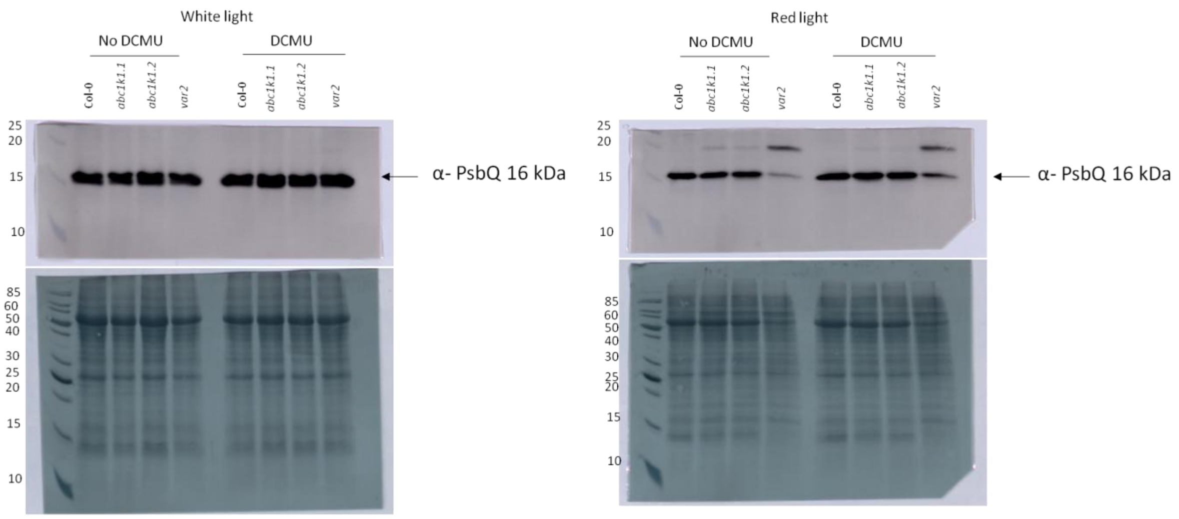

PsbQ Replicate 2

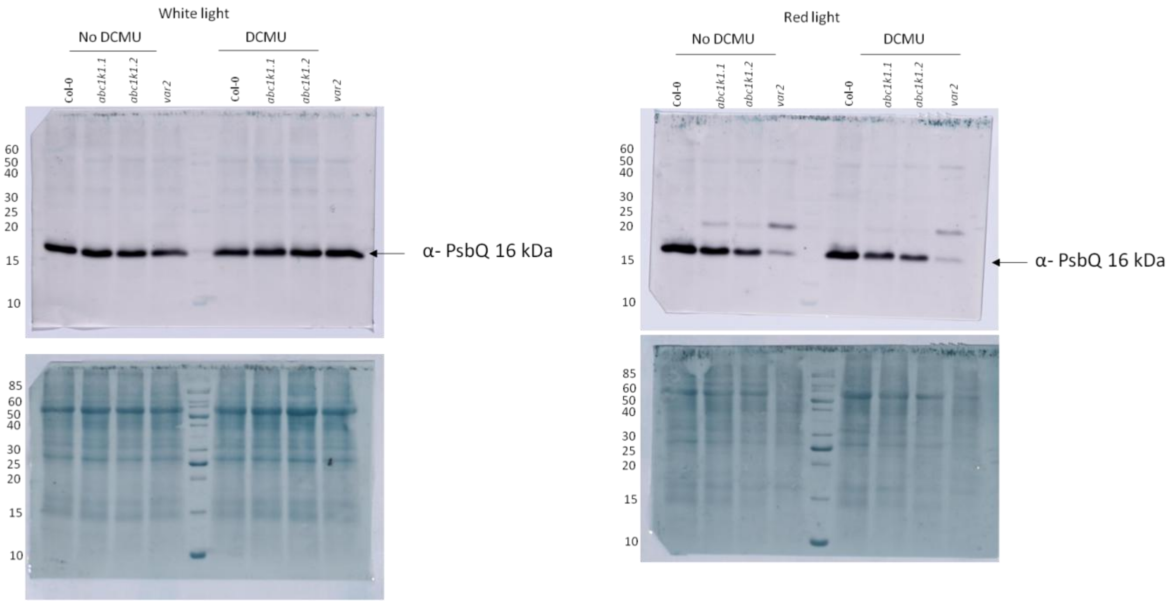

PsaN Replicate 1

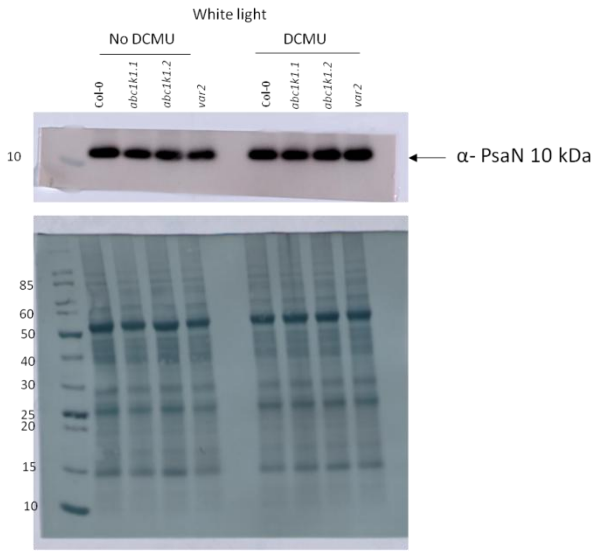

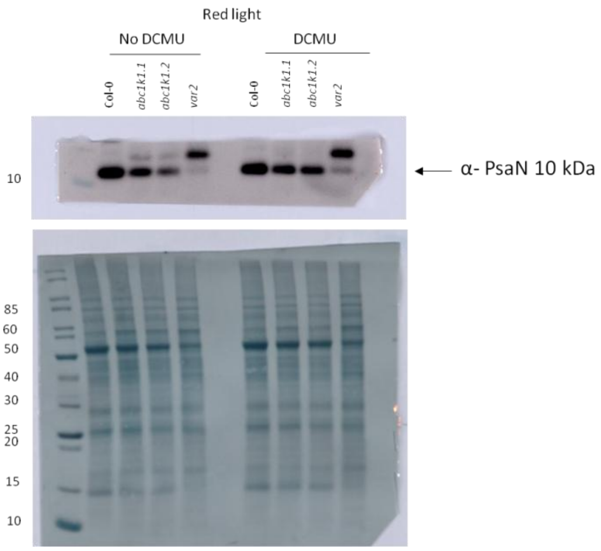

PsaN Replicate 2

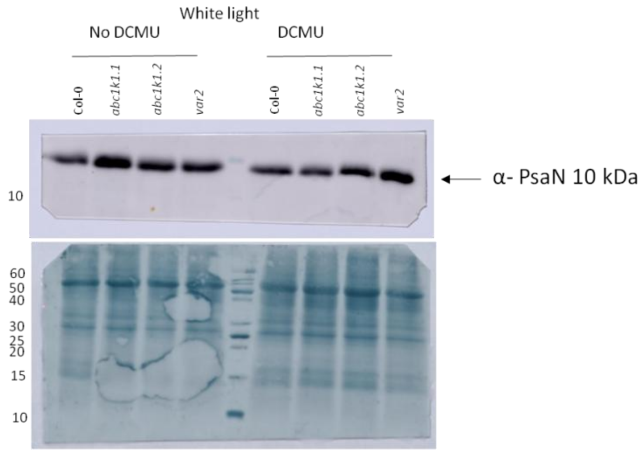

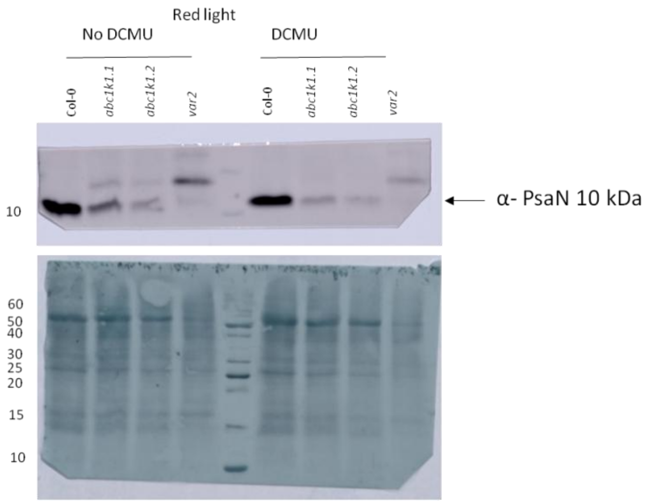

PsbA Replicate 1

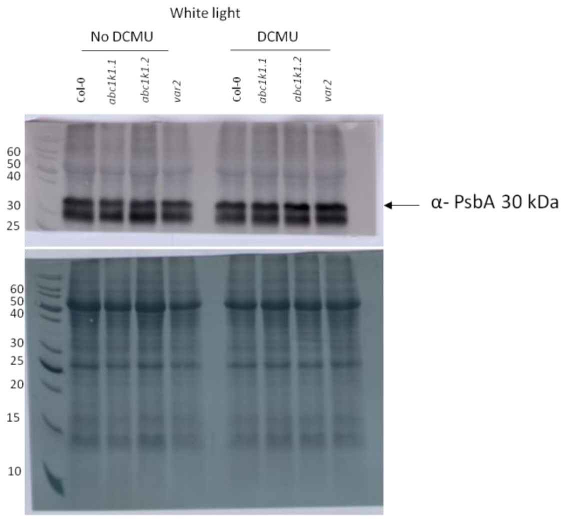

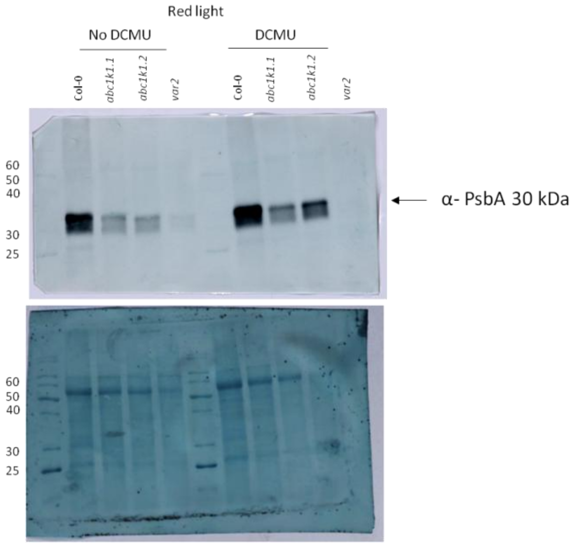

PsbA Replicate 2

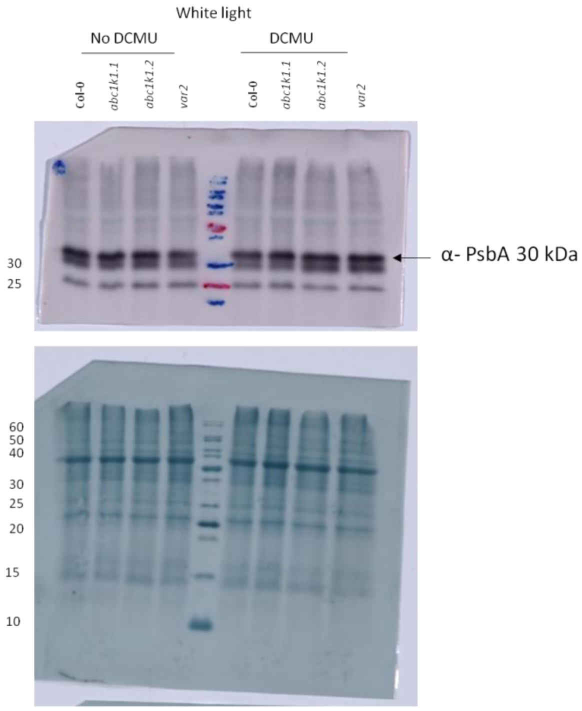

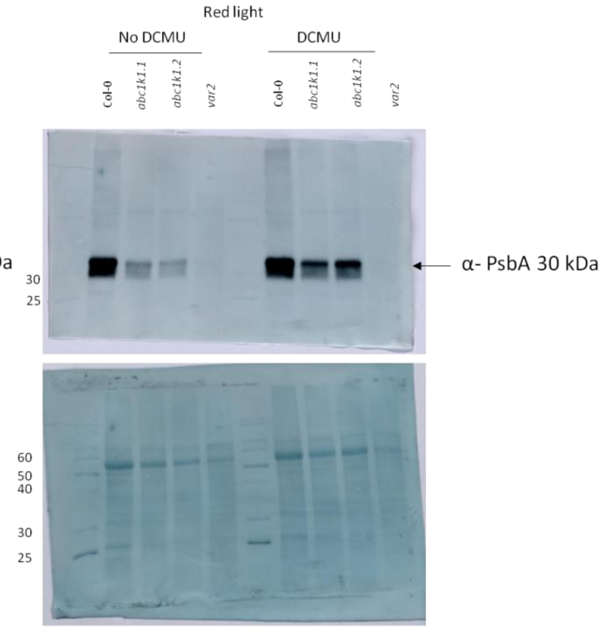

Supplement: Supplementary file 1 — Supplementary Information [file 42003_2025_7497_MOESM1_ESM.pdf]
