# Supplementary material for: Arabidopsis conditional photosynthesis mutants abc1k1 and var2 accumulate partially processed thylakoid preproteins and are defective in chloroplast biogenesis
Source: Commun Biol. 2025 Jan 22;8:111. doi: 10.1038/s42003-025-07497-y (PMC11754785; doi:10.1038/s42003-025-07497-y)
Supplement: Supplementary file 5 — Reporting Summary [file 42003_2025_7497_MOESM5_ESM.pdf]

nature portfolio

Corresponding author(s): Félix Kessler

Last updated by author(s): 14.12.2024

Reporting Summary

Nature Portfolio wishes to improve the reproducibility of the work that we publish. This form provides structure for consistency and transparency in reporting. For further information on Nature Portfolio policies, see our Editorial Policies and the Editorial Policy Checklist.

Statistics

For all statistical analyses, confirm that the following items are present in the figure legend, table legend, main text, or Methods section.

n/a	Confirmed	
☐	☒	The exact sample size (*n*) for each experimental group/condition, given as a discrete number and unit of measurement
☐	☒	A statement on whether measurements were taken from distinct samples or whether the same sample was measured repeatedly
☐	☒	The statistical test(s) used AND whether they are one- or two-sided<br>*Only common tests should be described solely by name; describe more complex techniques in the Methods section.*
☒	☐	A description of all covariates tested
☒	☐	A description of any assumptions or corrections, such as tests of normality and adjustment for multiple comparisons
☐	☒	A full description of the statistical parameters including central tendency (e.g. means) or other basic estimates (e.g. regression coefficient) AND variation (e.g. standard deviation) or associated estimates of uncertainty (e.g. confidence intervals)
☐	☒	For null hypothesis testing, the test statistic (e.g. *F*, *t*, *r*) with confidence intervals, effect sizes, degrees of freedom and *P* value noted<br>*Give P values as exact values whenever suitable.*
☒	☐	For Bayesian analysis, information on the choice of priors and Markov chain Monte Carlo settings
☒	☐	For hierarchical and complex designs, identification of the appropriate level for tests and full reporting of outcomes
☒	☐	Estimates of effect sizes (e.g. Cohen's *d*, Pearson's *r*), indicating how they were calculated

Our web collection on statistics for biologists contains articles on many of the points above.

Software and code

Policy information about availability of computer code

Data collection	For creation of the spectral library, data-dependent acquisition (DDA) was carried out using a standard TIMS PASEF method. The diaPASEF method used the same instrument parameters as the DDA methods (Bruker, Bremen, Germany).
Data analysis	Spectronaut 17.0.2, Perseus software package (Biognosys, Schlieren, Switzerland).

For manuscripts utilizing custom algorithms or software that are central to the research but not yet described in published literature, software must be made available to editors and reviewers. We strongly encourage code deposition in a community repository (e.g. GitHub). See the Nature Portfolio guidelines for submitting code & software for further information.

Data

Policy information about availability of data

All manuscripts must include a data availability statement. This statement should provide the following information, where applicable:

- Accession codes, unique identifiers, or web links for publicly available datasets
- A description of any restrictions on data availability
- For clinical datasets or third party data, please ensure that the statement adheres to our policy

All the data analysed are provided in supplementary tables. The proteomic data sets is accessible through the login page http://www.ebi.ac.uk/pride/login with the reviewer account details: username: reviewer_pxd050814@ebi.ac.uk , password: r226t2oO.

Research involving human participants, their data, or biological material

Policy information about studies with human participants or human data. See also policy information about sex, gender (identity/presentation), and sexual orientation and race, ethnicity and racism.

Reporting on sex and gender	n/a
Reporting on race, ethnicity, or other socially relevant groupings	n/a
Population characteristics	n/a
Recruitment	n/a
Ethics oversight	n/a

Note that full information on the approval of the study protocol must also be provided in the manuscript.

Field-specific reporting

Please select the one below that is the best fit for your research. If you are not sure, read the appropriate sections before making your selection.

☒ Life sciences ☐ Behavioural & social sciences ☐ Ecological, evolutionary & environmental sciences

For a reference copy of the document with all sections, see nature.com/documents/nr-reporting-summary-flat.pdf

Life sciences study design

All studies must disclose on these points even when the disclosure is negative.

Sample size	The sample size was determined empirically for each experiment (minimum of two independent samples) based on our experience with similar assays and sample sizes generally used by other investigators.
Data exclusions	No data were excluded from the analysis.
Replication	The experiment were replicated at least two times, the results were reproducible when the plants were not stressed before the experiment.
Randomization	When testing light condition the position of the plants of different genotypes was changed randomly in order to reduce any possible positioning effect.
Blinding	Was not required for this study.

Reporting for specific materials, systems and methods

We require information from authors about some types of materials, experimental systems and methods used in many studies. Here, indicate whether each material, system or method listed is relevant to your study. If you are not sure if a list item applies to your research, read the appropriate section before selecting a response.

Materials & experimental systems

n/a	Involved in the study	
☐	☒	Antibodies
☒	☐	Eukaryotic cell lines
☒	☐	Palaeontology and archaeology
☒	☐	Animals and other organisms
☒	☐	Clinical data
☒	☐	Dual use research of concern
☐	☒	Plants

Methods

n/a	Involved in the study	
☒	☐	ChIP-seq
☒	☐	Flow cytometry
☒	☐	MRI-based neuroimaging

Antibodies

Antibodies used	anti-D1 (PsbA) (Agrisera, AS05 084), anti-PsbB (Agrisera, AS04 038), anti-PsbO1 (Agrisera, AS14 2824), anti-PsbQ (Agrisera , AS06 142-16), anti-PsbP (Agrisera, AS06 142-23), anti-PsaD (Agrisera, AS09 461), anti-PsaN (Agrisera, AS06 109), anti-PetB (Agrisera, AS18 4169), anti-PetC (Agrisera, AS08 330), anti-plastocyanin (PC) (Agrisera , AS06 141), anti-Lhcb1 (Agrisera, AS09 522), anti-Lhca1

	(Agrisera, AS01 005), anti-TOC75 (Hiltbrunner et al., 2001), anti-Lhcb1-P (Agrisera, AS13 2704), anti-PsbA-P (Agrisera, AS13 2669). Primary antibodies were detected with horseradish peroxidase-conjµgated anti-rabbit (Merck, AP132P) antibodies
Validation	The primary antibodies were validated by the supplier on Arabidopsis thaliana total extracts.

Dual use research of concern

Policy information about dual use research of concern

Hazards

Could the accidental, deliberate or reckless misuse of agents or technologies generated in the work, or the application of information presented in the manuscript, pose a threat to:

No	Yes	
☒	☐	Public health
☒	☐	National security
☒	☐	Crops and/or livestock
☒	☐	Ecosystems
☒	☐	Any other significant area

Experiments of concern

Does the work involve any of these experiments of concern:

No	Yes	
☒	☐	Demonstrate how to render a vaccine ineffective
☒	☐	Confer resistance to therapeutically useful antibiotics or antiviral agents
☒	☐	Enhance the virulence of a pathogen or render a nonpathogen virulent
☒	☐	Increase transmissibility of a pathogen
☒	☐	Alter the host range of a pathogen
☒	☐	Enable evasion of diagnostic/detection modalities
☒	☐	Enable the weaponization of a biological agent or toxin
☒	☐	Any other potentially harmful combination of experiments and agents

Plants

Seed stocks	Arabidopsis thaliana wild-type is var. Columbia-0 (Col-0). Two ABC1K1 T-DNA insertion lines 342 (SALK_068628, 366 SALK_130499C) were obtained from the Nottingham Arabidopsis Stock 343 Centre (NASC, http://arabidopsis.info). The sps2 mutant was from Gilles Basset. The var2 344 (ftsh2) mutant was from Wataru Sakamoto.
Novel plant genotypes	n/a
Authentication	All genotypes have been verified in previous studies by others as well as us and are cited in the corresponding manuscript.
